# Integrated metabolite profiling and transcriptome analysis identify candidate genes involved in diterpenoid alkaloid biosynthesis in *Aconitum pendulum*

**DOI:** 10.3389/fpls.2025.1547584

**Published:** 2025-03-24

**Authors:** Ting Wang, Guoqing Xu, Zhaoyu Liu, Xiaoxia Ding, Liangting Wang, Liang Leng, Can Wang, Tong Xu, Yi Zhang

**Affiliations:** ^1^ Ethnic Medicine Academic Heritage Innovation Research Center, School of Ethnic Medicine, Chengdu University of Traditional Chinese Medicine, Chengdu, China; ^2^ Institute of Herbgenomics, Chengdu University of Traditional Chinese Medicine, Chengdu, China; ^3^ State Key Laboratory of Southwestern Chinese Medicine Resources, Chengdu University of Traditional Chinese Medicine, Chengdu, China; ^4^ School of Chinese Materia Medica, Tianjin University of Traditional Chinese Medicine, Tianjin, China

**Keywords:** *Aconitum pendulum*, transcriptome, metabolome, diterpenoid alkaloids biosynthesis, candidate genes

## Abstract

**Introduction:**

*Aconitum pendulum* is a well-known Tibetan medicine that possesses abundant diterpenoid alkaloids (DAs) with high medicinal value. However, due to the complicated structures of DAs and the associated challenges *in vitro* synthesis presents, plants like *Aconitum pendulum* remain the primary source for DAs.

**Methods:**

Given the underutilization of the *A. pendulum*, a thorough metabolomic and transcriptomic analysis was conducted on its flowers, leaves, and stems to elucidate the regulatory network underlying DA biosynthesis.

**Results:**

Metabolomic profiling (utilizing UPLC-QQQ-MS/MS) identified 198 alkaloids, of which 61 were DAs and the relative abundance of DAs was different among different tissues. Without a reference genome, we performed *de novo* assembly of the transcriptome of *A. pendulum*. We generated 181,422 unigenes, among which 411 candidate enzyme genes related to the DA synthesis pathway were identified, including 34 differentially expressed genes (DEGs). Through joint analysis of transcriptome and metabolome data, we found a correlation between the detected metabolite levels in various tissues and the expression of related genes. Specifically, it was found that ApCYP1, ApCYP72, and ApCYP256 may be related to turupellin accumulation, while ApBAHD9, ApBAHD10, ApBAHD12 positively associated with the accumulation of aconitine. Furthermore, our study also revealed that genes involved in the diterpene skeleton synthesis pathway tend to be highly expressed in flowers, whereas genes related to DA skeleton synthesis and their subsequent modifications are more likely to be highly expressed in leaf and stem tissues. Functional analysis of gene families identified 77 BAHD acyltransferases, 12 *O*-methyltransferases, and 270 CYP450 enzyme genes potentially involved in the biosynthesis of DAs. The co-expression network between metabolites and related genes revealed 116 significant correlations involving 30 DAs and 58 enzyme genes.

**Discussion:**

This study provides valuable resources for in-depth research on the secondary metabolism of *A. pendulum*, not only deepening our understanding of the regulatory mechanisms of DA biosynthesis but also providing valuable genetic resources for subsequent genetic improvement and metabolic engineering strategies.

## Introduction

*Aconitum pendulum* Busch, also known as “Tiebangchui”, is a perennial medicinal plant belonging to the *Aconitum* genus of the Ranunculaceae family. It is predominantly distributed in provinces such as Tibet, Qinghai, Sichuan, Shanxi, and Gansu in China (Li et al., 2022b). *A. pendulum* contains a variety of active ingredients, the most well-known of which is diterpenoid alkaloids (DAs). DAs have a significant analgesic and anti-inflammatory effect but also have a strong toxicity that can cause damage to the central nervous system, heart, and digestive system. Therefore, this plant is a typical medicinal species with therapeutic effects and potential toxicity (Ma et al., 2015; Tian et al., 2021). The traditional processing methods of Tibetan medicine, such as those involving Hezi decoction, Qingke wine, and Zanba, effectively reduce toxicity while preserving efficacy. The processed tubers of *A. pendulum* are commonly employed to treat various disorders, including fever, arthritis, rheumatic arthralgia, traumatic injury, furuncle, and tumors (Li et al., 2022b, 2023; Liu et al., 2023b).

DAs participate in the growth, development, and defense processes of plants through secondary metabolic pathways (Jan et al., 2021; Liu et al., 2023a). DAs can only be identified in specific aggregations of angiosperms, such as several genera within the families of Ranunculaceae, Rosaceae, and Escalloniaceae (Shen et al., 2020; Zhao et al., 2023). At present, many natural DAs are isolated from *Aconitum* plants, so *Aconitum* species can be used as a model for studying DAs (Shen et al., 2010; Zhao et al., 2018). Previous studies have shown that compared with traditional Chinese medicine *Aconitum carmichaelii*, the number and variety of alkaloids of the *A. pendulum* are significantly reduced. The relatively single chemical composition of *A. pendulum* provides an advantage for analyzing the biosynthetic pathway of DAs (Wang et al., 2016, 2019; Chen et al., 2021).

At present, the research on *A. pendulum* mainly focuses on the chemical composition and pharmacological activity, Owing to the scarcity of genomic information, the molecular mechanisms responsible for the biosynthesis and regulation of DAs in *A. pendulum* remain unclear (Kiss et al., 2013; Mao et al., 2021). DAs are classified into C_18_, C_19_, and C_20_-DAs based on carbon skeleton number, which share the same upstream pathway in plants (Tholl, 2015). It is speculated that DAs biosynthetic pathway first produces isopentenyl diphosphate (IPP) through Mevalonate (MVA) and methylerythritol (MEP) pathways. Then, IPP molecules are condensed by geranylgeranyl diphosphate synthase (GGPPS) to form diterpene precursor geranylgeranyl pyrophosphate (GGPP) (Cherney and Baran, 2011; Bergman et al., 2019). *Ent*-CPP synthases (CPS) convert GGPP to *ent*-copalyl diphosphate (*ent*-CPP), and subsequently produce *ent*-kaurene or *ent*-atiserene, which are the two most important diterpene frameworks (Yang et al., 2019; Mao et al., 2021). Further, *ent*-kaurene or *ent*-atiserene form C_20_-diterpenoid skeleton atisine and napeline under the action of aminotransferases (ATFs) (Zhao et al., 2009). The C_20_-type DAs thus formed might undergo Wagnere-Meerwein rearrangement and ring closure to ultimately form C_19_-type and C_18_-type DA skeletons. Structural modifications may be catalyzed by protein families such as cytochrome P450s (CYPs), *O*-methyltransferases (OMTs), and BAHD acyltransferases, forming structurally diverse DAs (Rai et al., 2017; Zhao et al., 2018; Xiao et al., 2019; Li et al., 2021).

The advancement of molecular sequencing technologies has significantly enhanced our understanding of the enzymes and metabolic pathways associated with the evolution of specialized secondary metabolites (Li et al., 2022a; Zhang et al., 2022; Zhao et al., 2023). Integrative transcriptome and metabolome data analysis have been effectively used to plant functional genes in the post-genomic age (Li et al., 2010). Based on the previous transcriptome study of *Aconitum* plants, it was found that the expression and content of DA biosynthesis genes were inconsistent in plant tissues, suggesting the existence of a source-sink relationship within plant tissues (Bai et al., 2022). Presently, the accumulation patterns of DAs within the tissues of *A. pendulum* and the underlying molecular mechanisms governing their biosynthesis remain poorly understood. In this work, we combined plant metabolomics, high-throughput transcriptome sequencing, *de novo* transcriptome assembly, and real-time reverse transcription PCR (qRT-PCR) of three tissues (flowers, leaves, and stems) of *A. pendulum* to initially elucidate the molecular mechanism underlying the differential accumulation of DAs (Liu et al., 2020; Song et al., 2023). Our findings present novel viewpoints regarding the biosynthetic regulation of DAs in different tissues of *A. pendulum* from a molecular perspective and offer valuable resources for the potential metabolic engineering of this crucial medicinal plant.

## Materials and methods

### Plant materials

The materials for the experiments were collected from the wild *A. pendulum* along Zhaha Road in Guide County, Qinghai Province, China, (101°25′4.53″ E, 36°21′52″ N, altitude 3681m) during August 2023. For flowers (AF), leaves (AL), and stems (AS), samples were gathered from three separate plants. A total of 9 sets of samples were obtained and promptly frozen in liquid nitrogen. They were kept at -80 °C before metabolite and RNA extraction experiments (Song et al., 2023). The plant was identified by Prof. Zhang of Chengdu University of Traditional Chinese Medicine as *Aconitum pendulum* Busch (**Supplementary Figure 1**).

### Sample extraction

The flowers, leaves, and stems of *A. pendulum* were freeze-dried in a vacuum by cryopreservation. A grinder (MM400, Retsch) was used to grind the samples for 1.5 minutes at 30 Hz into a powder. For each sample, 50mg of freeze-dried powder was mixed with 1.20 mL of 70% methanol solution pre-cooled to -20°C. The extracts underwent vortexing six times, with each time lasting for 30 seconds, at intervals of 30 minutes. After the extraction process, samples were centrifuged at 12,000 rpm for 3 minutes. The supernatant was filtered through a 0.22 μm microporous membrane and stored in a glass test tube for UPLC-QQQ-MS/MS analysis (Singh and Desgagné-Penix, 2017).

### Metabolomics determination

The ultra-performance liquid chromatography (ExionLC AD) and tandem mass spectrometry (MS/MS) were used to determine the content of alkaloids. The UPLC column used was an Agilent SB-C18 (1.80 µm, 2.10 mm × 100 mm). The solvent phase consisted of ultrapure water with 0.1% formic acid in acetonitrile (A) and 0.1% formic acid water (B). A gradient algorithm was run using 5% B as the starting condition. Within nine minutes, mobile phase B was raised to 95% with a linear gradient, and it was kept there for one minute. Mobile phase B was then maintained for 14 minutes after being modified to 5% in 10 minutes. A column was used to separate the samples at a flow rate of 0.35 mL/min and an injection volume of 2 μL (Duan et al., 2022). In this LC-MS/MS system, the principal parameters of the linear ion trap and triple quadrupole (QQQ) consisted of electrospray ionization at 550°C and mass spectrometry at 5500 V. The pressures of the ion source gas (GSI), gas (GSII), and curtain gas (CUR) were set at 50, 60, and 25 psi respectively, and the collision-induced ionization parameter was high. Mass spectrometry, quantification, and metabolite identification were carried out by Wuhan MetWare biotechnology company standard operating procedures (Che et al., 2020).

### Total RNA sequencing and *de novo* assembly

Total RNA was extracted from three tissues (flowers, leaves, and stems) from three individual plants and was treated as three replicates. The quality of the RNA samples was evaluated using a Nanodrop Photometer spectrophotometer and determined by agarose gel electrophoresis. mRNA was purified with oligo (dT) magnetic beads for further cDNA preparation. cDNA was synthesized by reverse transcription after RNA fragmentation, and amplified after connecting sequencing adapter to construct library. Nine transcriptome libraries of *A. pendulum* were constructed, after the library passed the quality inspection, the transcriptome of 9 samples was sequenced through the DNBSEQ platform. The Q20, Q30, and GC content in the clean data were calculated. Trinity software (v 2.15.1) was used to assemble the sequence, eliminate redundancy, and generate expression matrix after quantitative analysis to obtain transcript sequences. The completeness of the *de novo* assemblies was assessed using Benchmarking Universal Single-Copy Orthologs (BUSCO) (Simão et al., 2015).

### Gene function annotation and classification

To predict gene function, we used result files generated by eggNOG-mapper (v 2.1.12) for annotation. These annotations included Gene Ontology (GO) (http://geneontology.org/), Cluster of Orthologous Groups (COGs) (https://www.ncbi.nlm.nih.gov/COG/), and Kyoto Encyclopedia of Genes and Genomic Pathways (KEGG) (https://www.genome.jp/kegg/), We employed TBtools software to extract individual annotation files from these results. For the GO enrichment analysis, the GO ontology file go-basic.obo (https://geneontology.org/docs/download-ontology/) was loaded, and the enricher function was employed to conduct the GO enrichment analysis. BLAST (v 1.16.0) was utilized to compare the National Center for Biotechnology Information Non-Redundant Protein Sequence (Nr) (https://www.ncbi.nlm.nih.gov/refseq/about/nonredundantproteins/). Eventually, the annotated information on *A. pendulum* transcripts was obtained.

### Differential expression analysis of unigenes, and screening of enzyme genes involved in DA synthesis

Differential expression analysis between the two samples (AF vs AL, AF vs AS, AS vs AL) was performed using the DESeq2 package. Gene expression levels were calculated based on the transcripts per million (TPM), TPM = expression of transcript/(length of transcript (kb) × total expression (million)) values. Genes satisfying adjusted *p* < 0.05 were defined as differentially expressed genes (DEGs), which were subsequently functionally annotated via GO and KEGG pathway analyses. To identify enzyme genes associated with DA biosynthesis in the *A. pendulum*, we conducted BLAST analyses using enzyme genes related to DAs identified within the *Aconitum vilmorinianum* genome, in conjunction with transcriptome data from *A. pendulum*. Subsequently, by using the Hidden Markov Model (HMM) file of the Pfam ID (including PF09265, PF01397, PF03936, PF00067, PF001155, PF04864, PF00891, PF01596, PF02458) corresponding to the relevant enzyme gene downloaded from the Pfam database, The results from both BLAST and HMMER were intersected, and a threshold value of 1×10^-4^ was applied to screen for enzyme genes putatively involved in DA biosynthesis in *A. pendulum*. The DEGs and the enzyme genes with *p* < 0.05 were selected as candidate genes for subsequent joint analysis. Finally, TBtools (Chen et al., 2020) to create heatmaps of enzyme genes and metabolites.

### Phylogenetic analysis

Unigenes belonging to OMTs, CYPs, and BAHD acyltransferases were identified using BLAST software according to the characteristic domains. The nucleic acid sequences were converted into amino acid sequences by using the software seqkit (v2.8.2). Numerous functional protein sequences identified from other plant species were selected and individually compiled with OMTs, CYPs, and BAHD acyltransferases, and MEGA11 was used to construct phylogenetic trees (Tamura et al., 2021). The phylogenetic trees of OMTs, CYPs, and BAHD acyltransferases were constructed using the neighbor-joining method with the full-length amino acid sequences of the protein. The topology of phylogeny was evaluated by a bootstrap resampling analysis with 1,000 replicates. Finally, the interactive tree of life tool (https://itol.embl.de/) (Letunic and Bork, 2024) was used to enhance the visualization of the phylogenetic tree.

### Combined analysis based on metabolome and transcriptome

Association analysis of enzyme genes and secondary metabolites in *A. pendulum* to obtain candidate genes for the regulation of DA biosynthesis, after normalizing the expression levels of candidate genes and the accumulated DA contents in *A. pendulum*, the Pearson correlation algorithm method (Bishara and Hittner, 2012) was used to construct the regulatory networks of candidate genes and key metabolites (differential DAs and typical DAs in the biosynthetic pathways of DA). Correlation coefficients for co-expression analyses were calculated using R (v 4.2.1) software. Cytoscape software (v 3.10.1) was used to visualize the co-expression networks between enzyme genes and key metabolites (Kohl et al., 2011), with the following parameters: the absolute value of Pearson correlation coefficient > 0.8 and *p* < 0.05 (Zhang et al., 2021; Liu et al., 2023a).

### Quantitative real-time PCR verification analysis

To verify the accuracy of the expression levels obtained from RNA-Seq analysis, the widely accepted qRT-PCR technique was employed. Specific primers targeting selected genes were designed and used with TB Green Premix Ex Tap II (Takara, Dalian, China) according to the manufacturer’s protocol. qRT-PCR assay was performed using the QuantStudio 5 Real-Time PCR detection system (Applied Biosystems, Foster City, CA, USA). The specificity was confirmed through melt curve analysis, using the PP2A1 gene as an internal reference gene. The amplification reaction conditions were as follows: 95°C for 30 s, 40 cycles of 95°C for 5 s, and 60°C for 30 s. All qRT-PCR experiments were conducted with three biological replicates, and the relative expression levels were calculated based on the 2 ^−ΔΔCt^ method.

## Results

### The alkaloid metabolome analysis of *A. pendulum*

**Figure 1:**
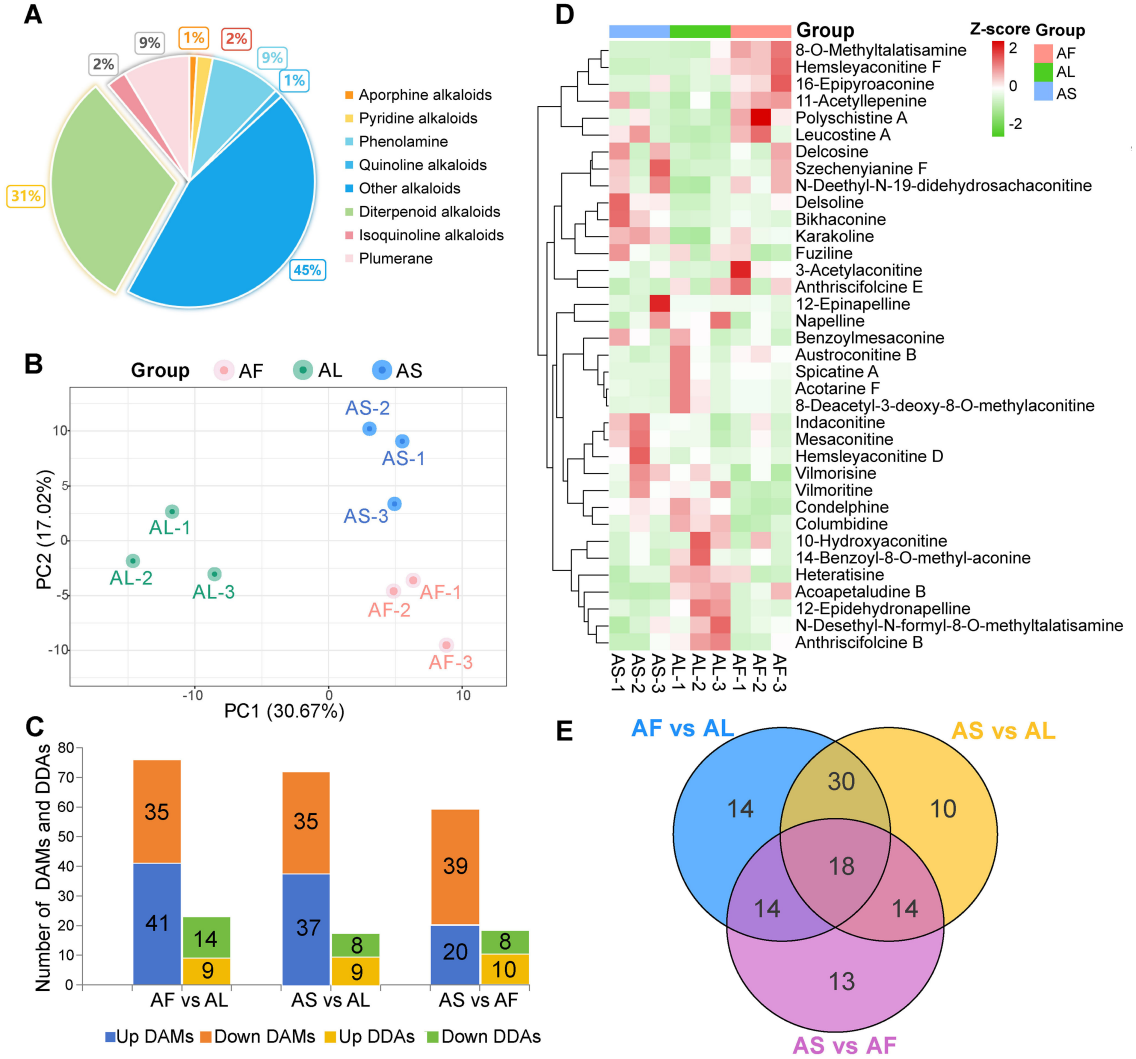
Metabolome analysis of flowers (AF), leaves (AL), and stems (AS) of *A*. *pendulum.* **(A)** Metabolite classification, diterpenoid alkaloids (DAs) account for 31% of the detected metabolites, **(B)** Principal component analysis (PCA) of nine samples from three tissues, **(C)** Number of up-regulated and down-regulated differential accumulation metabolites (DAMs) and differential diterpenoid alkaloids (DDAs) in different tissues, **(D)** Heatmap of DDAs based on hierarchical cluster analysis, **(E)** Venn diagram of DAMs.

A total of 198 alkaloid metabolites were identified from three *A. pendulum* tissues including 61 DAs (**Supplementary Figure 2**), 18 phenolamine, 17 plumerane, 5 isoquinoline alkaloids, 4 pyridine alkaloids, 2 quinoline alkaloids, 2 aporphine alkaloids, and 89 other alkaloids, the proportion of the number of alkaloids is shown in **Figure 1A**. The results of PCA analysis showed that PC1 was 30.67% and PC2 was 17.02%, indicating that the three tissue samples were significantly separated, but there was no significant difference within the group (**Figure 1B**), indicating the stability and reliability of the metabonomic test. Select VIP > 1 or fold change ≥ 2 and fold change ≤ 0.5 as the criteria, a total of 113 differential accumulation metabolites (DAMs) were screened, of which 36 were differential diterpenoid alkaloids (DDAs), detected 76, 72, and 59 DAMs in three groups (AF vs AL, AS vs AL, and AS vs AF, respectively) (**Figure 1C**). 41 up-regulated and 35 down-regulated DAMs were identified between AF vs AL, these metabolites mainly included 23 differential diterpenoid alkaloids (DDAs). In the “AS vs AL” group, there were 72 DAMs, of which 37 were up-regulated DAMs and 35 down-regulated DAMs, including 17 DDAs. 20 up-regulated and 39 down-regulated DAMs were identified between AS vs AF, including 18 DDAs. The heatmap of hierarchical clustering showed that the accumulation of DDAs in different tissues of *A. pendulum* varied considerably (**Figure 1D**), The number of DDAs in stems and leaves was higher than in flowers. In the flowers, compounds such as hemsleyaconitine F, 8-*O*-methyltalatisamine, 11-acetyllepenine, 16-epipyroaconine, and polyschistine A were significantly enriched. In the leaves, acoapetaludine B, heteratisine, and 12-epidehydronapelline are more abundant. Finally, delsoline, mesaconitine, and karakoline are more enriched in the stems. In the three comparison groups, there were fourteen, ten, and thirteen DAMs with single differences, and there were eighteen common DAMs, of which two were DDAs, respectively polyschistine A and hemsleyaconitine F (**Figure 1E**).

The KEGG database was used for functional pathway annotation analysis of DAMs (**Supplementary Figure 3**). All the differential metabolites were found to be annotated to metabolism, genetic information processing, and environmental information processing. The top three metabolic pathways were the metabolic pathways, biosynthesis of secondary metabolites, and tryptophan metabolism. The results of KEGG enrichment showed that there were differences in enrichment metabolic pathways and signal transduction pathways among the three groups. Differential metabolites were mainly concentrated in tryptophan metabolism, phenylalanine, tyrosine and tryptophan biosynthesis, benzoxazinoid biosynthesis, and biosynthesis of various plant secondary metabolites. In the AF vs AL and AS vs AF groups, the main differential pathways were tryptophan metabolism and metabolic pathways, respectively. In AS vs AL groups, the primary differential pathways are tryptophan metabolism, benzoxazinoid biosynthesis, and biosynthesis of secondary metabolites. It is speculated that these different metabolic pathways are one of the reasons for the difference in metabolites in different tissues.

### Transcriptome sequencing and gene functional annotation

**Figure 2:**
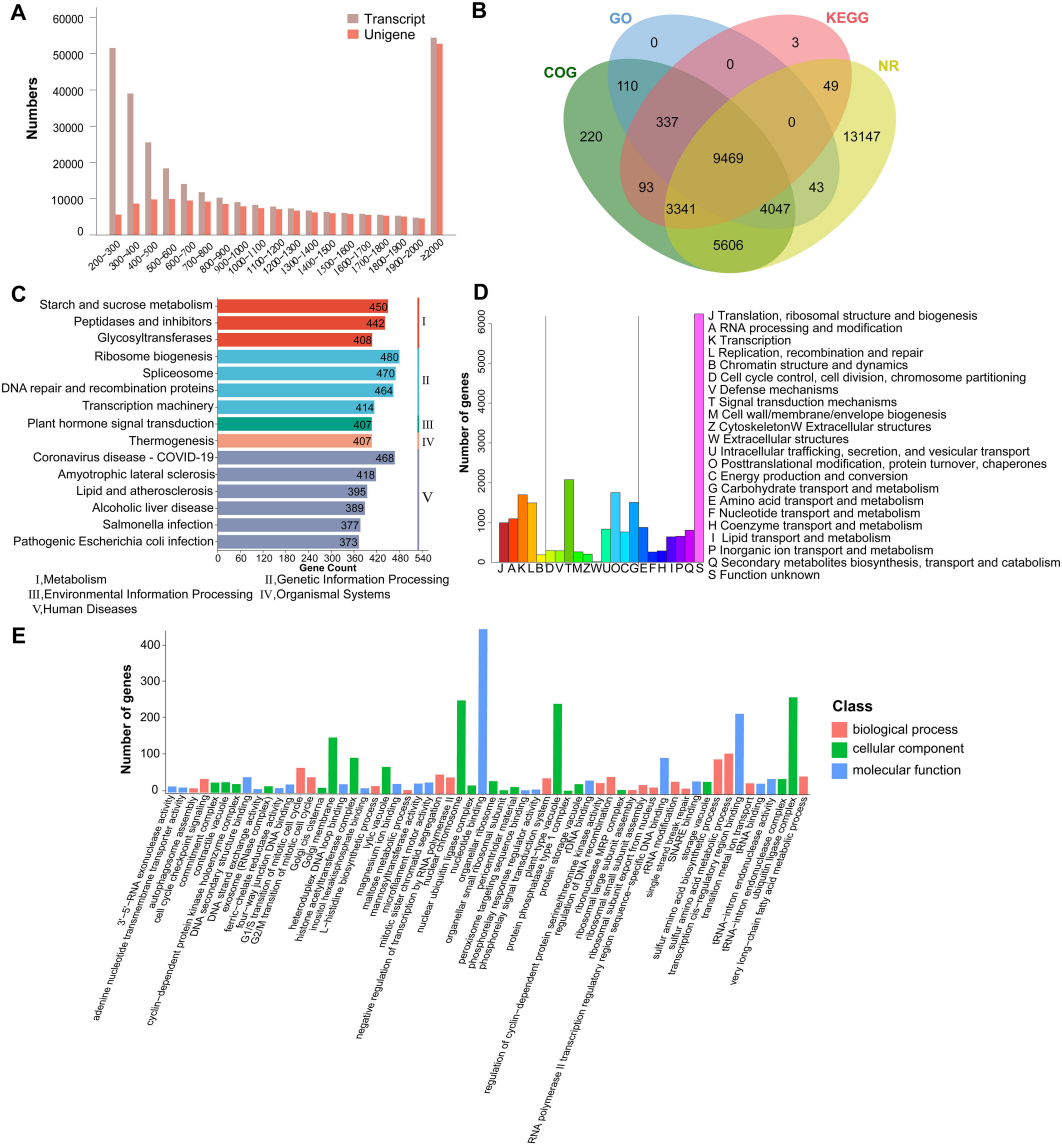
Diagrams of the database annotation results and assembly for *A*. *pendulum* unigenes. **(A)** Length distribution of the assembled transcripts and unigenes, **(B)** Venn diagrams of annotations unigene numbers from the four major databases, **(C)** KEGG database annotations, **(D)** COG database annotations, **(E)** Gene Ontology classification of the assembled unigenes.

The transcriptome of three tissues of *A. pendulum* was analyzed. The raw reads obtained by the RNA sequencing were processed. A total of 21.95 – 30.47 million clean reads were obtained after removing low-quality and incorrect reads from raw reads, the average clean data of each sample reached over 6 Gb, the Q30 base was 97% and above, the quality of the reads of different tissues was provided in **Supplementary Table 1**. The completeness (complete single-copy BUSCOs 29.9% and complete duplicated BUSCOs 67.9%) of the assembly was assessed using BUSCO (v5.8.2) (**Supplementary Figure 4**), these data indicate that the assembly outcomes are favorable and suitable for subsequent research. The total number, minimum length, maximum length, mean length, N50, and N90 of the transcripts and unigenes are summarized in **Table 1**. The N50 of the transcript was 1,974 bp, the N90 of the transcript was 471 bp, its minimum length was 191 bp, its maximum length was 17,073 bp, and its mean length was 1,165 bp. These results showed the assembly of high-quality transcriptomes in this research. To get a unique representative transcript for a single unigene, the longest transcript was taken as a single unigene for each gene regardless of splice variants. A total of 181,422 unigenes were assigned from a total of 298,191 assembled transcripts, of which 112,371 (61.94%) were longer than 1,000 bp. The length distributions of the transcripts and unigenes are shown in **Figure 2A**.

**Table 1:** Summary statistics of the RNA-seq results.

	Transcript	Unigene	Unigene_gt1000*
Total number	298,191	181,422	112,371
Mean length (bp)	1,165	1,628	2,254
Min length (bp)	191	197	1,001
Max length (bp)	17,073	17,073	17,073
N50 (bp)	1,974	2,216	2,484
N90 (bp)	471	831	1,312

^*unigene_gt1000: Genes length of at least 1000bp.^

The annotation results from four databases for the transcriptome sequencing data of *A. pendulum* showed that 36,465 unigenes were successfully annotated, including 35,702 unigenes in the Nr database, 13,292 unigenes in the KEGG database, 23,223 unigenes in the COG database, and 14,006 unigenes in the GO database. Among these unigenes, 9,469 were annotated in all four databases (**Figure 2B**). Based on KEGG, 13,292 (7.3%) unigene sequences were assigned to 369 KEGG pathways, and the unigenes were mapped to five hierarchical pathways: metabolism, genetic information processing, environmental information processing, organismal systems, and human diseases (**Figure 2C**). The pathways involving the largest number were ribosome biogenesis, it is worth noting that 74 belong to terpenoid backbone biosynthesis. In the COG annotation, unigenes were classified into 22 functional categories: the largest group was S (function unknown, 6,242, 26.87%), followed by T (signal transduction mechanisms, 2,074, 8.93%), and the smallest category was W (extracellular structures, 14, 0.06%) (**Figure 2D**). Moreover, 13,292 (7.32%) unigenes were divided into three functional GO categories. Among categories, regulation of signal transduction and obsolete cellular carbohydrate metabolic process were the largest subcategory in the biological process category, chromosome was the most enriched term in the cellular component category, and glycosyltransferase activity was the top term in the molecular function category (**Figure 2E**).

### Analysis of differentially expressed genes

**Figure 3:**
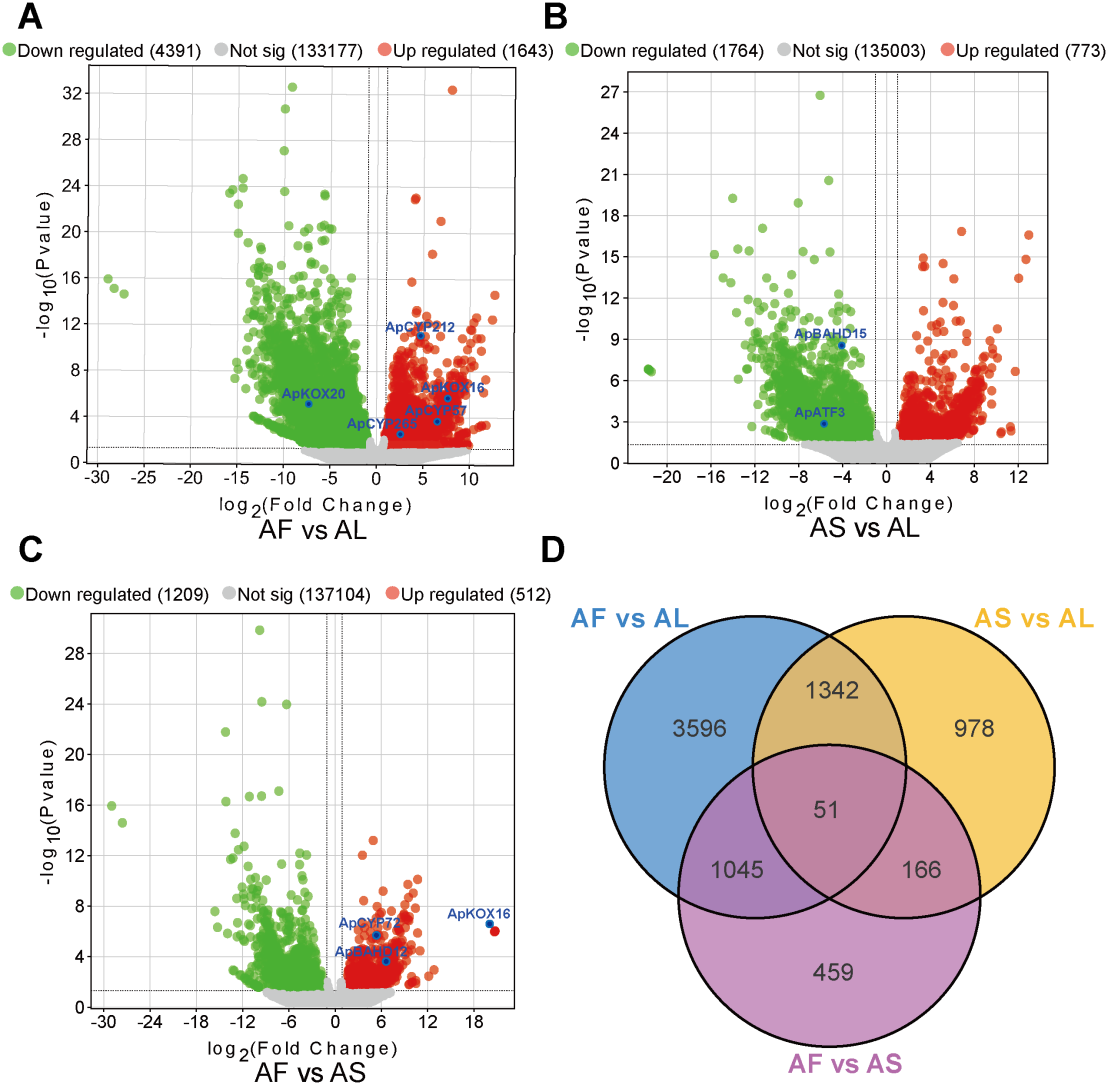
Analysis of differentially expressed genes (DEGs) in different tissues of *A*. *pendulum*. **(A)** Volcano plots of DEGs in AF vs AL, **(B)** Volcano plots of DEGs in AS vs AL, **(C)** Volcano plots of DEGs in AF vs AS, **(D)** Venn diagram of DEGs identified in different comparison groups.

We analyzed the genes enriched in the metabolic pathway of DAs and screened DEGs in three tissues using an adjusted *P* < 0.05 as the criterion. 6,034, 2,537, and 1,721 DEGs were detected in the three comparison groups of AF vs AL, AS vs AL, and AF vs AS groups. The largest number of DEGs were identified between AF and AL, there were 1,643 up-regulated DEGs, including genes such as ApKOX16, ApCYP57, ApCYP212, and ApCYP265. and 4,391 down-regulated DEGs, including ApKOX20. In the comparison between AS and AL, 773 were up-regulated and 1,764 were down-regulated, among which both ApATF3 and ApBAHD15 were down-regulated. Lastly, AF vs AS revealed the lowest number of differentially expressed genes DEGs: 512 were up-regulated, including ApKOX16, ApCYP72, and ApBAHD12, while 1,209 were down-regulated. Volcano plots of the different comparisons were shown in **Figures 3A–C**. Venn diagrams were built to depict the distributions and relationships of DEGs among paired comparisons and 51 DEGs were demonstrated to be commonly changed (**Figure 3D**), and the heat map of the correlation coefficients for three tissue samples is presented in **Supplementary Figure 5**.

To gain a better understanding of the biological functions of these DEGs, GO and KEGG enrichment analyses were performed. AF vs AL DEGs were enriched in plastid thylakoid, chloroplast thylakoid, obsolete thylakoid part, and cytoskeleton, revealing their roles in photosynthesis, mechanical support, and protection; AS vs AL DEGs were enriched in plant-type cell wall, apoplastm, photosynthesis, and abaxial cell fate specification; AF vs AS DEGs were enriched in GO terms mainly related to post-embryonic plant morphogenesis, root morphogenesis, and post-embryonic plant organ development, which indicated that these DEGs may be directly involved in the development and morphological construction of their respective organs (**Supplementary Figure 6**). The DEGs were enriched in 332 KEGG pathways, and the significant pathways for each pairwise comparison are presented in **Supplementary Figure 7**. The KEGG pathways such as glyoxylate and dicarboxylate metabolism, zeatin biosynthesis, two-component system, and pentose and glucuronate interconversions repeatedly appeared in various comparison groups, involving multiple aspects including plant growth and development, environmental adaptation, and signal transduction. This indicates that DEGs associated with these pathways are expressed in all three tissues but with differences in their expression levels.

### Candidate genes encoding enzymes involved in DA biosynthesis

To identify candidate enzyme genes associated with DA biosynthesis, we screened the annotated transcriptome assembly of *A. pendulum* to search for homologs corresponding to the eight enzyme gene families in the proposed biosynthetic pathway to retain the strongest candidates for the subsequent analysis. 411 enzyme genes may be involved in DA synthesis (**Supplementary Table 2**). Among them, the DEGs with adjusted *p* < 0.05 are listed in **Supplementary Table 3**, while the genes with *p* < 0.05 are listed in **Supplementary Table 4**. Meanwhile, a heatmap of gene expression levels of *p* < 0.05 and DEGs is presented in **Supplementary Figure 8**. The eight gene families encompass GGPPS, CPS, and KS genes, which are likely involved in the biosynthesis of diterpenoids; KOX and ATF genes, which may contribute to the formation of the DA skeleton; and CYPs, OMTs, and BAHD acyltransferase genes, which play roles in modifying the DA skeleton. The number of the enzyme genes and the DEGs is shown in **Table 2**.

**Table 2:** The unigenes related to DA biosynthesis.

Abbreviation of gene	Name of the gene	Enzyme no.	Number of genes	Number of DEGs
GGPPS	Geranylgeranyl diphosphate synthase, type II	K13789	2	0
CPS	*ent*-Copalyl diphosphate synthase	K04120	5	0
KS	*ent*-Kaurene synthase	K04121	12	0
CYP450(KOX)	*ent*-Kaurene oxidase	K04122	20	3
ATF	Aminotransferase	K00826	33	1
OMT	*O*-methyltransferase	K22091	12	1
BAHD	BAHD acyltransferase	K19747	77	9
CYP450	Cytochrome P450	K14338	250	20

### Integrated analysis of the candidate genes and metabolites of DA biosynthesis pathway in *A. pendulum*

**Figure 4:**
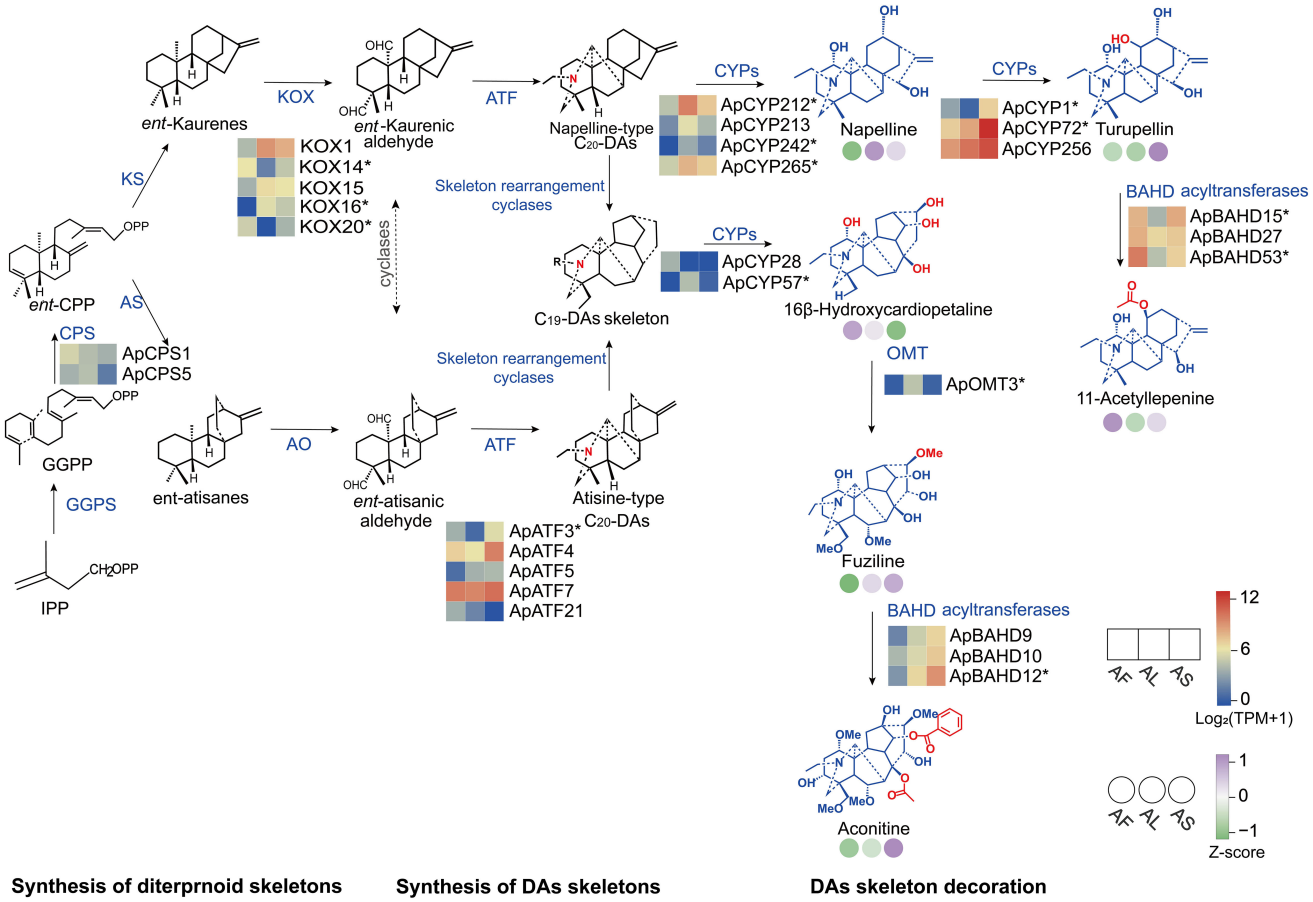
DA biosynthetic pathway in the three tissues of *A. pendulum*. The heat map shows the flowers (AF), leaves (AL), and stems (AS) from left to right. The color grid represents the candidate enzyme genes (*p* < 0.05, gene* represents DEGs) expression level according to the color scale. The color circle represents the relative content levels of typical DAs in the biosynthetic pathways. IPP, isoprenoid; GGPP, geranylgeranyl pyrophosphate; GGPPS, geranylgeranyl pyrophosphate synthase, CPS, *ent*-copalyl diphosphate synthase; KS, *ent*-kaurene synthase; KOX, *ent*-Kaurene oxidase; AS, *ent*-atisane synthase; AO, *ent*-atisane oxidases; ATF, L-serine aminotransferases; CYPs, Cytochrome P450 monooxygenase; and OMT, *O*-methyltransferase.

To identify candidate genes potentially involved in the biosynthetic pathway of DA in *A. pendulum*, the Pearson correlation coefficient is used to measure the association between the content differences of typical DAs in different tissues and the expression patterns of related enzyme genes (*p* < 0.05) in the biosynthetic pathway of DAs (**Supplementary Table 5**). Based on these analysis results, candidate enzyme genes strongly correlated with the typical DAs detected by metabolomics in the DA synthesis pathway were selected, and a possible biosynthetic pathway for *A. pendulum* was constructed. The biosynthetic pathway of DAs can be divided into three stages: diterpene precursor formation, DA skeleton formation, and diterpenoid alkaloid skeleton modification (**Figure 4**). The unigene dataset identified most enzyme genes involved in DA biosynthesis, including CPSs, ATFs, CYPs, OMTs, and BAHD acyltransferases. Genes involved in the synthesis of terpenoid skeleton are expressed at relatively higher levels in flowers. In comparison, genes involved in the synthesis of DA skeleton and subsequent modification are expressed at higher levels in leaves and stems. Specifically, the napelline-type C_20_ skeleton may be catalyzed by ApCYP212, ApCYP213, ApCYP242, and ApCYP265, which are relatively highly expressed in leaves and stems, to produce napelline. Furthermore, high expression in stems may facilitate the catalysis of hydroxylation by ApCYP1, ApCYP72, and ApCYP256 to generate turupellin. ApBAHD15, ApBAHD27, and ApBAHD53 may be associated with the accumulation of 11-acetyllepenine. Additionally, ApCYP28 and ApCYP57 may participate in the modification of the C_19_-DA skeleton to produce 16*β*-hydroxycardiopetaline. It is speculated that ApOMT3 catalyzes methylation to generate fuziline. Furthermore, ApBAHD9, ApBAHD10, and ApBAHD12, significantly expressed in stems, may be involved in the synthesis of aconitine. There was a significant correlation between the relative content of DAs and different tissue expression patterns of related enzyme genes, but fewer gene expressions encoded may not perfectly align with metabolite accumulation, indicating potential additional functions of the pathway.

### Identification and analysis of gene families involved in DA skeleton modification

We obtained 12 unigenes encoding OMT from transcriptome data. Phylogenetic tree analysis revealed that six ApOMT enzyme genes (ApOMT1, ApOMT2, ApOMT3, ApOMT4, ApOMT5, ApOMT6) clustered together with other Flavonoid 4-*O*-methyltransferases enzyme genes, among which ApOMT1 shared high sequence homology with PsOMT3. The remaining six ApOMT enzyme genes (ApOMT7, ApOMT8, ApOMT9, ApOMT10, ApOMT11, ApOMT12 clustered with various known caffeoyl-CoA 3-*O*-methyltransferase (CCoAOMT) (**Supplementary Figure 9**).

CYPs catalyze the specific hydroxylation of terpene compounds. In the current research, 270 CYP genes have been identified from the transcriptome and named as ApCYP genes. Based on the clustering of the identified ApCYP genes in *A.pendulum* and P450s in *Arabidopsis thaliana* in the phylogenetic tree, we have divided the CYPs family into 9 clans. It was found that most P450s involved in terpene skeleton decoration catalysis belong to the CYP71 family, followed by the CYP86 family. KOXs may catalyze the conversion of *ent*-kaurene to active intermediates, and 19 annotated KOX genes formed an independent branch within the CYP701 clade of the CYP71 family (**Supplementary Figure 10**).

BAHD acyltransferase is the key terminal enzyme that catalyzes the synthesis of ester groups, indicating that BAHD acyltransferase may be involved in the formation of *Aconitum* toxicity. Cluster analysis was performed on 77 ApBAHD acyltransferase genes and BAHD acyltransferase genes with identified functions. The results showed that the BAHD acyltransferase family could be divided into different classes, including I, II, III, IV, V (V_a_ and V_b_), and unknown. Eight genes are potentially classified as belonging to subfamily III. Additionally, eleven genes are potentially classified as belonging to the V_a_ subfamily. Among them, ApBAHD26 and ApBAHD76 cluster on the same branch with NtBEBT and CbBEBT, suggesting a high degree of phylogenetic relatedness and potentially similar biological functions. These BAHD acyltransferases may contribute to the acute toxicity of DAs (**Supplementary Figure 11**).

### Correlation analysis of metabolites and related genes reveals the relationship involved in DA biosynthesis candidate genes

**Figure 5:**
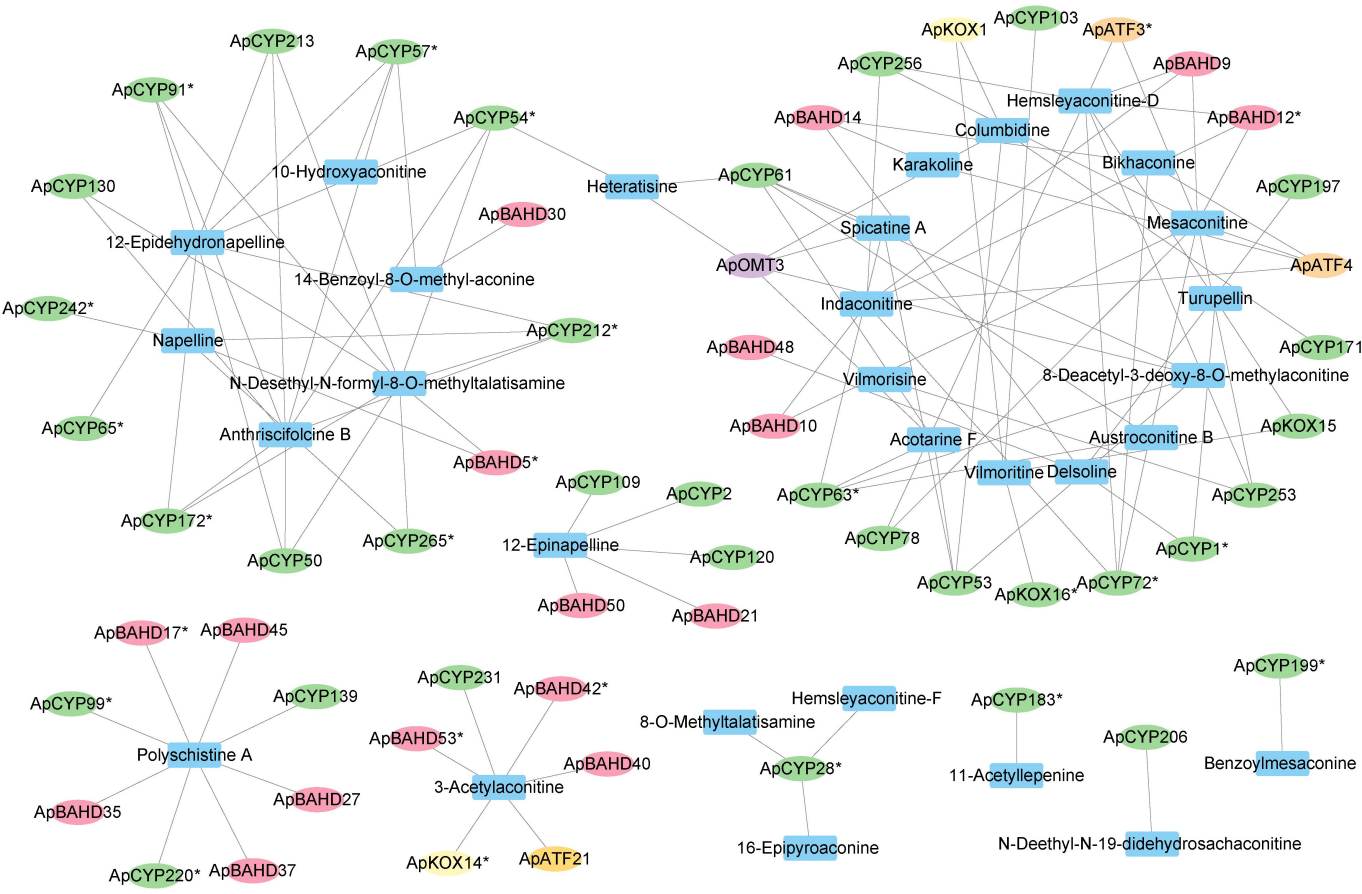
Visualization of the connectivity network of DA-related candidate enzyme genes (*p* < 0.05, gene* represents DEGs) and key DAs.

We performed an integrated analysis of the transcriptome and metabolome of the flowers, leaves, and stems of *A. pendulum*. Pearson correlation coefficient > 0.8, and *p* < 0.05 were calculated between each set of variables to establish a gene-metabolite regulatory network (**Figure 5**). Notably, 58 candidate enzyme genes exhibited strong correlation coefficients with 30 key DAs, yielding 116 interaction networks between metabolites and candidate genes (**Supplementary Table 6**). Specifically, ApCYP99, ApCYP139, ApCYP220, ApBAHD17, ApBAHD27, ApBAHD35, ApBAHD37, and ApBAHD45 displayed strong correlations with polyschistine A; ApKOX14, ApATF21, ApCYP231, ApBAHD40, ApBAHD42 and ApBAHD53 showed a unique strong correlation with 3-acetylaconitine; ApCYP2, ApCYP109, ApCYP120, ApBAHD21, ApBAHD50 correlated with 12-epinapelline, while ApCYP183 correlated solely with 11-acetyllepenine and ApCYP199 correlated solely with benzoylmesaconine, 24 ApCYP genes demonstrated strong correlations with numerous DAs. The relationships between enzyme genes and key DAs within this network corroborate their potential functions in the DA pathway. Enzyme genes catalyze the modification of functional groups in DAs.

### Validation of gene expression profiles by qRT-PCR

**Figure 6:**
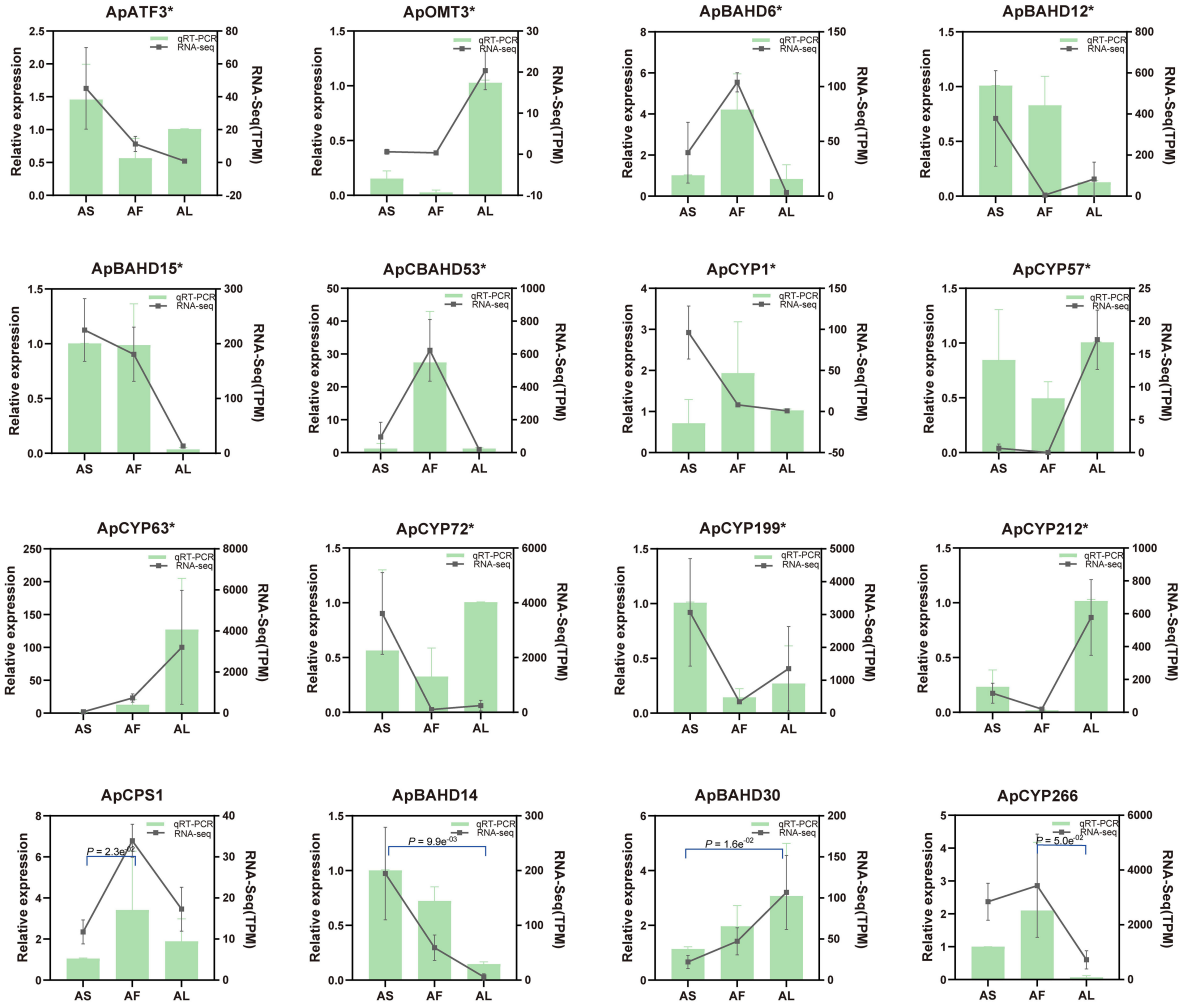
qRT-PCR validation of selected enzyme genes in *A. pendulum*. The lines represent the expression level TPM of the potential 16 genes (*p* < 0.05, gene* represents DEGs) involved in the DA biosynthetic pathway (right y-axis); the columns represent the relative expression of qRT-PCR from three biological replicates (left y-axis).AS, stems; AF, flowers; AL, leaves.

qRT-PCR was performed to demonstrate the reliability of the transcriptional data. 16 genes related to DA biosynthesis in *A. pendulum* were randomly selected. Among them, 12 were DEGs, Additionally, ApCPS1, ApBAHD14, ApBAHD30, and ApCYP266 are genes with *p* < 0.05, and the primer information for these enzyme genes and the internal reference gene is listed in **Supplementary Table 7**. The results demonstrate that the expression trends observed in qRT-PCR and transcriptome analysis are largely similar, and these genes exhibit marked differential expression across various tissues. The results of the transcriptome data have been verified (**Figure 6**).

## Discussion

Due to the lack of genomic data for *A. pendulum*, the assembly and annotation of transcriptome data present challenges. Therefore, it is necessary for us to utilize the Trinity software tool for *de novo* transcriptome assembly. However, our results may suffer from an overestimation of the number of annotated transcripts and unigenes (Bai et al., 2022). Through the assembly of the transcriptome of *A. pendulum*, 181,422 unigenes and 298,191 complete transcripts were generated, but with the criterion for identifying DEGs being set at adjusted *p* < 0.05, we found that 6,034, 2,537, and 1,721 DEGs were detected in the three comparison groups of AF vs AL, AS vs AL, and AF vs AS, respectively. The number of DEGs in each group was less than 5% of the total unigene counts. From a biological perspective, the number of DEGs between two tissues should not be very low, so we suspect that the use of Trinity-based *de novo* assembly to obtain 181,422 unigenes may have introduced issues in subsequent quantification, resulting in the number of identified DEGs being far less than the actual number. Therefore, in subsequent analyses, we included both the DEGs and genes with *p* < 0.05 for further investigation. To identify the genes directly involved in DA synthesis in the *A. pendulum*, a more accurate but relatively smaller set of candidate genes was obtained. In the last decades, herb-genomics has seen remarkable progress with the successful creation of high-quality assemblies for medicinal plants (Leng et al., 2024; Yang et al., 2024). Therefore, a high-quality genome assembly of plants such as *A. pendulum* should be carried out as soon as possible, which would facilitate a more thorough understanding of the DA biosynthesis process.

Diterpenoids originate from the common precursor IPP, biosynthesized through two intersecting but independent pathways: the MVA and MEP (Walker and Croteau, 2000). Here, we focused on genes involved in the formation of the diterpene and DA skeletons after IPP synthesis, as well as the genes post-modification of the skeleton of DA. A total of 411 enzyme genes encoding catalytic enzymes involved in DA biosynthesis were identified, and 77 candidate enzyme genes (*p* < 0.05) were screened out, among which 34 were DEGs. Our study found that differential genes involved in diterpenoid skeleton formation were relatively highly expressed in flowers, while those involved in diterpenoid alkaloid skeleton formation and modification were relatively highly expressed in leaves and stems. Meanwhile, Studies on *A. vilmorinianum* and *A. kusnezoffii* also suggested a possible source-sink relationship between aerial and subterranean tissues, with DAs potentially synthesized in the aerial parts (leaves and stems) and then gradually transported and accumulated in the subterranean parts (Bai et al., 2022; Zhao et al., 2023; Zhou et al., 2024). Our research further supports this hypothesis. In addition, by comparison, it was found that the number of DEGs in AF vs AL is significantly higher than that in AF vs AS, as well as in AS vs AL. It is speculated that when plants need to grow, photosynthesis in the green part is enhanced, and the primary and secondary metabolism is vigorous, which led to an increase in the number of DEGs in AF vs AL.

Plants frequently synthesize specialized metabolites in response to both biotic and abiotic environments, encompassing alkaloids, terpenoids, and phenylpropanoids (Feng et al., 2022). These compounds synthesized from plants can be used as drug precursors and it is expected to become targeted drugs with medicinal value after chemical modification or transformation (Chen et al., 2024; Wang et al., 2025). Among these, the DAs produced by *A. pendulum* are recognized as significant bioactive compounds due to their important medicinal values in anti-inflammatory and analgesic activities. Previously, researchers conducted metabolomic analyses on different parts of seven *Aconitum* species, determining the quantity and types of alkaloids in *A. pendulum*. They found that the quantity and types of alkaloids in *A. pendulum* were significantly reduced compared to those in the traditional Chinese medicine *Aconitum kusnezoffii*. 66 DAs (including C_18_, C_19_, and C_20_-DAs) were identified from *Aconitum* species, whereas only 14 DAs (C_19_ and C_20_-DAs) were identified in *A. pendulum* (Chen et al., 2021). This finding indicates that the relatively single chemical composition of *A. pendulum* plants offers advantages for analyzing the biosynthetic pathway of DA. Through a broad-target metabolomic analysis, we identified 198 alkaloids, one-third of which are DAs. Most of these alkaloids are derived from the C_19_-DA scaffold through substituent variations, followed by napelline-type C_20_-DAs. However, C_18_-DAs have not been detected in the *A. pendulum*. Based on differences in substituents at the C-8 and C-14 positions, these DAs are classified into three categories: diester diterpenoid alkaloids, monoester diterpenoid alkaloids (MDA), and non-esterified diterpenoid alkaloids (NDA) (Qiu et al., 2021). All kinds of DAs were distributed in different tissues, but their contents were different. Additionally, KEGG analysis of the differentially enriched genes in three tissues revealed enrichment in tryptophan metabolism and phenylalanine metabolism pathways, suggesting potential synergistic effects with the biosynthesis of DA.

The medicinal part of *Aconitum* plants mainly consists of roots., while the aerial parts are often discarded, resulting in significant resource wastage. Studies have demonstrated that the stems and leaves (JY) of *A. carmichaelii* account for approximately 40% of the plant’s total biomass, indicating the substantial value of the aerial parts (Zhou et al., 2024). Our transcriptome results are consistent with the metabolic profiling, which shows a relatively high abundance of differential diterpenoid alkaloid metabolites in the stems and leaves. This further validates the medicinal potential of the stems and leaves. Furthermore, JY exhibits comparable anti-inflammatory and analgesic effects to the fibrous roots, while the acute toxicity of JY was also low (He et al., 2018). These findings suggest that the aerial parts, including the stems and leaves of *A. pendulum*, can serve as alternative medicinal portions. The utilization and exploration of these parts can provide new avenues for phytotherapy and the conservation of medicinal resources.

If the modification of DAs occurs in a step-wise manner, structurally diverse intermediate or transitional products may directly contribute to the diversity and complexity of DAs. The varying combinations of types, numbers, and positions of functional groups greatly enrich the structural diversity of DAs. The reason for the diverse types of DAs lies in the modification by related enzyme genes, primarily including CYP450s, which primarily provide hydroxyl groups; *O*-methyltransferases responsible for methylation; and BAHD acyltransferases, which primarily provide acyl groups. Most of the CYPs associated with terpene modification belong to the CYP71 family, which is involved in the metabolism of most specialized compounds (Mau and Croteau, 2006). Further research can be conducted to determine whether CYP71 members play a role in catalyzing the C-8 and C-14 hydroxylation of DAs (Zhao et al., 2023). Additionally, The introduction of amino groups into the skeleton of DAs is also a crucial step in their biosynthesis. The diterpene skeleton may introduce amino groups through transamination enzymes or by conjugating with N-containing compounds (such as arginine or ornithine derivatives), with the mechanism possibly involving CYPs-mediated oxidative amination or condensation with amino acid side chains. OMT modifies alkaloids to produce products with specific methylation patterns. A study has found that the methylation rate of C_19_-DAs in *A. carmichaelii* is relatively high (48.1%), suggesting that methyltransferases may be involved in catalyzing the formation of *O*-methylation in DAs. OMT-mediated methylation enhances the solubility, stability, and bioactivity of DAs, aiding in defense and adaptation to environmental changes (Lam et al., 2024). BAHD acyltransferases constitute a unique superfamily of genes in plants that acylate secondary metabolites. The biosynthesis of DAs involves extensive acyl modification, which is also a crucial factor in the formation of toxicity and activity of DAs. Acyl or benzoyl modifications exist at various positions such as C-3, C-8, and C-14. The acetyltransferases and benzoyltransferases involved in alkaloid synthesis are concentrated in subfamily III and subfamily V_a_ of the BAHD acyltransferase family, it has been reported that enzymes in III subfamily primarily utilize acetyl-CoA as the acyl donor and various alcohol compounds as substrates to participate in alkaloid biosynthesis (Bayer et al., 2004), and enzymes in V_a_ subfamily can utilize benzoyl-CoA as an acyl donor to participate in the biosynthesis of benzoates (Walker and Croteau, 2000), it is speculated that these BAHD acyltransferase genes may play a role in the biosynthesis of benzoate esters in alkaloids (Zhao et al., 2009). Pearson correlation analysis was employed to evaluate the significance of the correlation between the expression levels of diterpenoid pathway-related candidate genes and DA content in tissues. The identification of enzyme genes strongly correlated with key metabolites has laid a foundation for the cloning of candidate enzyme genes in *A. pendulum*. Currently, the biosynthesis of DAs faces enormous challenges. It is necessary to further clarify the nitrogen sources and catalytic enzymes, and how environmental factors or developmental stages affect the expression of synthetic genes. Additionally, reconstructing the pathway through heterologous expression (such as in yeast) to achieve industrial production is also a crucial task.

*In situ* biosynthesis and transport phenomena influence the accumulation of bioactive compounds within medicinal plant tissues (Jiang et al., 2021). Comprehensive analyses integrating transcriptomics and metabolomics have been extensively utilized to delineate the relationship between the dynamic changes of secondary metabolites and the differential expression of corresponding genes in the biosynthetic pathways of bioactive components in various medicinal plants (Wan et al., 2024). This combined analytical approach is beneficial for gaining new insights into the accumulation and biosynthesis of DAs in *A. pendulum*.

## Conclusion

This study integrates metabolite and transcriptome analyses to investigate the DA biosynthetic regulatory network of *A. pendulum*. We identified 61 specific DA metabolites and 411 genes potentially involved in DA biosynthesis. Correlations were observed between some candidate enzyme genes and DA accumulation, leading to the hypothesis that DA synthesis is transported from flowers to leaves and stems. Further investigation into the gene families primarily involved in the modification of complex and diverse diterpenoid alkaloid functional groups was conducted to explore their primary functions. Finally, based on Pearson correlation coefficient analysis, we constructed a co-expression network consisting of 58 genes highly correlated with 30 DAs, which exert positive regulatory effects on these metabolites. The transcriptome and metabolome data provide valuable resources for studying genes involved in biosynthesis. The data set can provide a reference for DA metabolism, molecular identification, molecular breeding, and other further studies.

## Data Availability

The datasets presented in this study can be found in online repositories. The names of the repositories and accession number can be found at the Genome Sequence Archive (GSA), accession number: CRA023287 by using following link: https://ngdc.cncb.ac.cn/search/?dbId=gsa&q=CRA023287.

## References

[B1] Bai S. Sartagnuud S. Wang T. Bao G. Bao S. Ao W. (2022). De nove transcriptome sequencing identifies genes involved in aconitine-type alkaloids biosynthesis in Aconitum kusnezoffii Reichb. Pharmacol. Res. - Modern Chin. Med. 2, 100063. doi: 10.1016/j.prmcm.2022.100063

[B2] Bayer A. Ma X. Stöckigt J. (2004). Acetyltransfer in natural product biosynthesis—-functional cloning and molecular analysis of vinorine synthase. Bioorganic Medicinal Chem. 12, 2787–2795. doi: 10.1016/j.bmc.2004.02.029 15110860

[B3] Bergman M. E. Davis B. Phillips M. A. (2019). Medically useful plant terpenoids: biosynthesis, occurrence, and mechanism of action. Molecules 24, 3961. doi: 10.3390/molecules24213961 31683764 PMC6864776

[B4] Bishara A. J. Hittner J. B. (2012). Testing the significance of a correlation with nonnormal data: Comparison of Pearson, Spearman, transformation, and resampling approaches. psychol. Methods 17, 399–417. doi: 10.1037/a0028087 22563845

[B5] Che P. Liu J.-S. Qi Y.-D. Qiang T.-Y. Song Y.-C. Wei X.-P.. (2020). Simultaneous determination of six major isosteroidal alkaloids in Beimu by UPLC-ELSD. Zhongguo Zhong Yao Za Zhi 45, 1393–1398. doi: 10.19540/j.cnki.cjcmm.20191223.201 32281353

[B6] Chen C. Chen H. Zhang Y. Thomas H. R. Frank M. H. He Y.. (2020). TBtools: an integrative toolkit developed for interactive analyses of big biological data. Mol. Plant 13, 1194–1202. doi: 10.1016/j.molp.2020.06.009 32585190

[B7] Chen L. Lai C. Mao L. Yin B. Tian M. Jin B.. (2021). Chemical constituents in different parts of seven species of Aconitum based on UHPLC-Q-TOF/MS. J. Pharm. Biomed. Anal. 193, 113713. doi: 10.1016/j.jpba.2020.113713 33160222

[B8] Chen Y. Yang J. Wang C. Wang T. Zeng Y. Li X.. (2024). Aptamer-functionalized triptolide with release controllability as a promising targeted therapy against triple-negative breast cancer. J. Exp. Clin. Cancer Res. 43, 207. doi: 10.1186/s13046-024-03133-5 39054545 PMC11270970

[B9] Cherney E. C. Baran P. S. (2011). Terpenoid-alkaloids: their biosynthetic twist of fate and total synthesis. Israel J. Chem. 51, 391–405. doi: 10.1002/ijch.201100005 PMC4508874 26207071

[B10] Duan Y. Liu X. Wu J. You J. Wang F. Guo X.. (2022). Transcriptomic and metabolic analyses reveal the potential mechanism of increasing steroidal alkaloids in Fritillaria hupehensis through intercropping with Magnolia officinalis. Front. Plant Sci. 13. doi: 10.3389/fpls.2022.997868 PMC9585282 36275508

[B11] Feng M. Chen C. Qu-Bie J. Qu-Bie A. Bao X. Cui Q.. (2022). Metabolome and transcriptome associated analysis of sesquiterpenoid metabolism in Nardostachys jatamansi. Front. Plant Sci. 13. doi: 10.3389/fpls.2022.1041321 PMC9746346 36523614

[B12] He Y.-N. Ou S.-P. Xiong X. Pan Y. Pei J. Xu R.-C.. (2018). Stems and leaves of *Aconitum carmichaelii* Debx. as potential herbal resources for treating rheumatoid arthritis: Chemical analysis, toxicity and activity evaluation. Chin. J. Natural Medicines 16, 644–652. doi: 10.1016/S1875-5364(18)30104-3 30269841

[B13] Jan R. Asaf S. Numan M. Lubna Kim K.-M. (2021). Plant secondary metabolite biosynthesis and transcriptional regulation in response to biotic and abiotic stress conditions. Agronomy 11, 968. doi: 10.3390/agronomy11050968

[B14] Jiang C. Fei X. Pan X. Huang H. Qi Y. Wang X.. (2021). Tissue-specific transcriptome and metabolome analyses reveal a gene module regulating the terpenoid biosynthesis in Curcuma wenyujin. Ind. Crops Products 170, 113758. doi: 10.1016/j.indcrop.2021.113758

[B15] Kiss T. Orvos P. Bánsághi S. Forgo P. Jedlinszki N. Tálosi L.. (2013). Identification of diterpene alkaloids from Aconitum napellus subsp. firmum and GIRK channel activities of some Aconitum alkaloids. Fitoterapia 90, 85–93. doi: 10.1016/j.fitote.2013.07.010 23876370

[B16] Kohl M. Wiese S. Warscheid B. (2011). “Cytoscape: software for visualization and analysis of biological networks,” in Data Mining in Proteomics: From Standards to Applications. Eds. Hamacher M. Eisenacher M. Stephan C. (Humana Press, Totowa, NJ). doi: 10.1007/978-1-60761-987-1_18 21063955

[B17] Lam L. P. Y. Lui A. C. W. Bartley L. E. Mikami B. Umezawa T. Lo C. (2024). Multifunctional 5-hydroxyconiferaldehyde *O* -methyltransferases (CAldOMTs) in plant metabolism. J. Exp. Bot. 75, 1671–1695. doi: 10.1093/jxb/erae011 38198655

[B18] Leng L. Xu Z. Hong B. Zhao B. Tian Y. Wang C.. (2024). Cepharanthine analogs mining and genomes of Stephania accelerate anti-coronavirus drug discovery. Nat. Commun. 15, 1537. doi: 10.1038/s41467-024-45690-5 38378731 PMC10879537

[B19] Letunic I. Bork P. (2024). Interactive Tree of Life (iTOL) v6: recent updates to the phylogenetic tree display and annotation tool. Nucleic Acids Res. 52, W78–W82. doi: 10.1093/nar/gkae268 38613393 PMC11223838

[B20] Li Y.-G. Mou F.-J. Li K.-Z. (2021). *De novo* RNA sequencing and analysis reveal the putative genes involved in diterpenoid biosynthesis in Aconitum vilmorinianum roots. 3 Biotech. 11, 96. doi: 10.1007/s13205-021-02646-6 PMC7840826 33520582

[B21] Li X. Park N. I. Xu H. Woo S.-H. Park C. H. Park S. U. (2010). Differential expression of flavonoid biosynthesis genes and accumulation of phenolic compounds in common buckwheat (*Fagopyrum esculentum*). J. Agric. Food Chem. 58, 12176–12181. doi: 10.1021/jf103310g 21062042

[B22] Li C.-Y. Sha M.-X. Pei Z.-Q. Zhou Z. Tang C. Liu Y.. (2023). Dynamic variations in the chemical constituents of Tiebangchui stir-fried with Zanba by integrating UPLC-Q-TOF-MS based metabolomics and DESI-MSI. Arabian J. Chem. 16, 104957. doi: 10.1016/j.arabjc.2023.104957

[B23] Li C.-Y. Yang L. Liu Y. Xu Z.-G. Gao J. Huang Y.-B.. (2022a). The sage genome provides insight into the evolutionary dynamics of diterpene biosynthesis gene cluster in plants. Cell Rep. 40, 111236. doi: 10.1016/j.celrep.2022.111236 35977487

[B24] Li C.-Y. Zhou Z. Xu T. Wang N.-Y. Tang C. Tan X.-Y.. (2022b). Aconitum pendulum and Aconitum flavum: A narrative review on traditional uses, phytochemistry, bioactivities and processing methods. J. Ethnopharmacology 292, 115216. doi: 10.1016/j.jep.2022.115216 35331875

[B25] Liu J. Han L. Li G. Zhang A. Liu X. Zhao M. (2023a). Transcriptome and metabolome profiling of the medicinal plant Veratrum mengtzeanum reveal key components of the alkaloid biosynthesis. Front. Genet. 14. doi: 10.3389/fgene.2023.1023433 PMC9895797 36741317

[B26] Liu J. Leng L. Liu Y. Gao H. Yang W. Chen S.. (2020). Identification and quantification of target metabolites combined with transcriptome of two rheum species focused on anthraquinone and flavonoids biosynthesis. Sci. Rep. 10, 20241. doi: 10.1038/s41598-020-77356-9 33219248 PMC7679448

[B27] Liu X. Tao H. Tian R. Huang W. Zhang T. Liu Y.. (2023b). Hezi inhibits Tiebangchui-induced cardiotoxicity and preserves its anti-rheumatoid arthritis effects by regulating the pharmacokinetics of aconitine and deoxyaconitine. J. Ethnopharmacology 302, 115915. doi: 10.1016/j.jep.2022.115915 36375646

[B28] Ma L. Gu R. Tang L. Chen Z.-E. Di R. Long C. (2015). Important poisonous plants in tibetan ethnomedicine. Toxins 7, 138–155. doi: 10.3390/toxins7010138 25594733 PMC4303819

[B29] Mao L. Jin B. Chen L. Tian M. Ma R. Yin B.. (2021). Functional identification of the terpene synthase family involved in diterpenoid alkaloids biosynthesis in Aconitum carmichaelii. Acta Pharm. Sin. B 11, 3310–3321. doi: 10.1016/j.apsb.2021.04.008 34729318 PMC8546855

[B30] Mau C. J. D. Croteau R. (2006). Cytochrome P450 oxygenases of monoterpene metabolism. Phytochem. Rev. 5, 373–383. doi: 10.1007/s11101-006-9008-2 PMC2901147 20622990

[B31] Qiu Z.-D. Zhang X. Wei X.-Y. Chingin K. Xu J.-Q. Gao W.. (2021). Online discovery of the molecular mechanism for directionally detoxification of Fuzi using real-time extractive electrospray ionization mass spectrometry. J. Ethnopharmacology 277, 114216. doi: 10.1016/j.jep.2021.114216 34044076

[B32] Rai M. Rai A. Kawano N. Yoshimatsu K. Takahashi H. Suzuki H.. (2017). *De novo* RNA sequencing and expression analysis of aconitum carmichaelii to analyze key genes involved in the biosynthesis of diterpene alkaloids. Molecules 22, 2155. doi: 10.3390/molecules22122155 29206203 PMC6150021

[B33] Shen Y. Liang W.-J. Shi Y.-N. Kennelly E. J. Zhao D.-K. (2020). Structural diversity, bioactivities, and biosynthesis of natural diterpenoid alkaloids. Nat. Prod. Rep. 37, 763–796. doi: 10.1039/D0NP00002G 32129397

[B34] Shen Y. Zuo A. Jiang Z. Zhang X. Wang H. Chen J. (2010). Five new C _19_ -diterpenoid alkaloids from *aconitum hemsleyanum* . Helv. Chimica Acta 93, 482–489. doi: 10.1002/hlca.200900228

[B35] Simão F. A. Waterhouse R. M. Ioannidis P. Kriventseva E. V. Zdobnov E. M. (2015). BUSCO: assessing genome assembly and annotation completeness with single-copy orthologs. Bioinformatics 31, 3210–3212. doi: 10.1093/bioinformatics/btv351 26059717

[B36] Singh A. Desgagné-Penix I. (2017). Transcriptome and metabolome profiling of Narcissus pseudonarcissus ‘King Alfred’ reveal components of Amaryllidaceae alkaloid metabolism. Sci. Rep. 7, 17356. doi: 10.1038/s41598-017-17724-0 29229969 PMC5725579

[B37] Song Y. Zhang G. Chen N. Zhang J. He C. (2023). Metabolomic and transcriptomic analyses provide insights into the flavonoid biosynthesis in sea buckthorn (Hippophae rhamnoides L.). LWT 187, 115276. doi: 10.1016/j.lwt.2023.115276

[B38] Tamura K. Stecher G. Kumar S. (2021). MEGA11: molecular evolutionary genetics analysis version 11. Mol. Biol. Evol. 38, 3022–3027. doi: 10.1093/molbev/msab120 33892491 PMC8233496

[B39] Tholl D. (2015). “Biosynthesis and biological functions of terpenoids in plants,” in Biotechnology of Isoprenoids. Eds. Schrader J. Bohlmann J. (Springer International Publishing, Cham), 63–106. doi: 10.1007/10_2014_295 25583224

[B40] Tian M. Chen L.-L. Jin B.-L. Guo J. Ge H. Zhao X.. (2021). Transcriptome analysis to identify genes involved in the biosynthesis of aconitines in *Aconitum pendulum* . Acta Pharm. Sin. 56, 3353–3361. doi: 10.16438/j.0513-4870.2021-0975

[B41] Walker K. Croteau R. (2000). Taxol biosynthesis: Molecular cloning of a benzoyl- CoA:taxane 2α- *O* -benzoyltransferase cDNA from *Taxus* and functional expression in *Escherichia coli* . Proc. Natl. Acad. Sci. U.S.A. 97, 13591–13596. doi: 10.1073/pnas.250491997 11095755 PMC17620

[B42] Wan L. Huang Q. Li C. Yu H. Tan G. Wei S.. (2024). Integrated metabolome and transcriptome analysis identifies candidate genes involved in triterpenoid saponin biosynthesis in leaves of Centella asiatica (L.) Urban. Front. Plant Sci. 14. doi: 10.3389/fpls.2023.1295186 PMC10811118 38283979

[B43] Wang B. Dong J. Ji J. Yuan J. Wang J. Wu J.. (2016). Study on the Alkaloids in Tibetan Medicine *Aconitum pendulum* Busch by HPLC–MS ^n^ Combined with Column Chromatography. J. Chromatogr Sci. 54, 752–758. doi: 10.1093/chromsci/bmw002 26896350 PMC4890449

[B44] Wang J. Meng X.-H. Chai T. Yang J.-L. Shi Y.-P. (2019). Diterpenoid alkaloids and one lignan from the roots of aconitum pendulum busch. Nat. Prod. Bioprospect. 9, 419–423. doi: 10.1007/s13659-019-00227-y 31728851 PMC6872700

[B45] Wang C. Xu H. Chen Y. Li X. Chen H. Liu J.. (2025). Hydroxyl-based acid-hypersensitive acetal ester bond: Design, synthesis and the application potential in nanodrugs. Eur. J. Medicinal Chem. 283, 117153. doi: 10.1016/j.ejmech.2024.117153 39681042

[B46] Xiao H. Zhang Y. Wang M. (2019). Discovery and engineering of cytochrome P450s for terpenoid biosynthesis. Trends Biotechnol. 37, 618–631. doi: 10.1016/j.tibtech.2018.11.008 30528904

[B47] Yang Y. Hu P. Zhou X. Wu P. Si X. Lu B.. (2019). Transcriptome analysis of aconitum carmichaelii and exploration of the salsolinol biosynthetic pathway. Fitoterapia 140, 45. doi: 10.21203/rs.2.10189/v1 31698060

[B48] Yang H. Wang C. Zhou G. Zhang Y. He T. Yang L.. (2024). A haplotype-resolved gap-free genome assembly provides novel insight into monoterpenoid diversification in *Mentha suaveolens ‘* Variegata’. Horticulture Res. 11, uhae022. doi: 10.1093/hr/uhae022 PMC10925848 38469381

[B49] Zhang X. Lin S. Peng D. Wu Q. Liao X. Xiang K.. (2022). Integrated multi-omic data and analyses reveal the pathways underlying key ornamental traits in carnation flowers. Plant Biotechnol. J. 20, 1182–1196. doi: 10.1111/pbi.13801 35247284 PMC9129081

[B50] Zhang A. Zheng J. Chen X. Shi X. Wang H. Fu Q. (2021). Comprehensive analysis of transcriptome and metabolome reveals the flavonoid metabolic pathway is associated with fruit peel coloration of melon. Molecules 26, 2830. doi: 10.3390/molecules26092830 34068821 PMC8126211

[B51] Zhao P.-J. Gao S. Fan L.-M. Nie J.-L. He H.-P. Zeng Y.. (2009). Approach to the biosynthesis of atisine-type diterpenoid alkaloids. J. Nat. Prod. 72, 645–649. doi: 10.1021/np800657j 19275222

[B52] Zhao D. Shen Y. Shi Y. Shi X. Qiao Q. Zi S.. (2018). Probing the transcriptome of Aconitum carmichaelii reveals the candidate genes associated with the biosynthesis of the toxic aconitine-type C19-diterpenoid alkaloids. Phytochemistry 152, 113–124. doi: 10.1016/j.phytochem.2018.04.022 29758520

[B53] Zhao D. Zhang Y. Ren H. Shi Y. Dong D. Li Z.. (2023). Multi-omics analysis reveals the evolutionary origin of diterpenoid alkaloid biosynthesis pathways in *Aconitum* . JIPB 65, 2320–2335. doi: 10.1111/jipb.13565 37688324

[B54] Zhou Y. Qu C. Yan H. Chu T. Wu J. Kang Q.. (2024). Unlocking the hidden potential: Enhancing the utilization of stems and leaves through metabolite analysis and toxicity assessment of various parts of Aconitum carmichaelii. J. Ethnopharmacology 323, 117693. doi: 10.1016/j.jep.2023.117693 38176669

