# Supplementary material for: Integrated metabolite profiling and transcriptome analysis identify candidate genes involved in diterpenoid alkaloid biosynthesis in Aconitum pendulum
Source: Front Plant Sci. 2025 Mar 24;16:1547584. doi: 10.3389/fpls.2025.1547584 (PMC11973281; doi:10.3389/fpls.2025.1547584)
Supplement: Supplementary file 1 [file DataSheet1.docx]

Supplementary Material


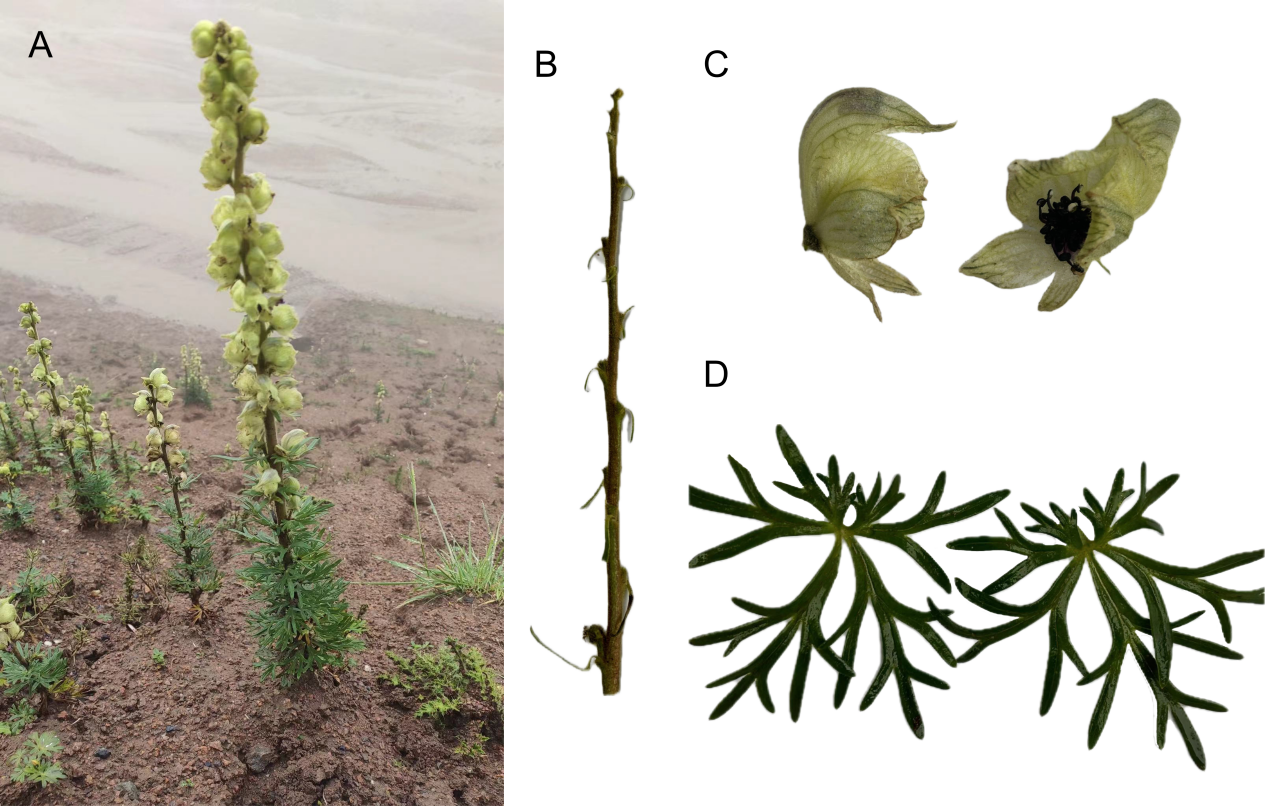


**Supplementary Figure 1.** The plant of *A.pendulum.* **(A)** The plants and environment. **(B)** The stem of the adult plant. **(C)** The flower of the adult plant. **(D)** The leaves of the adult plant.


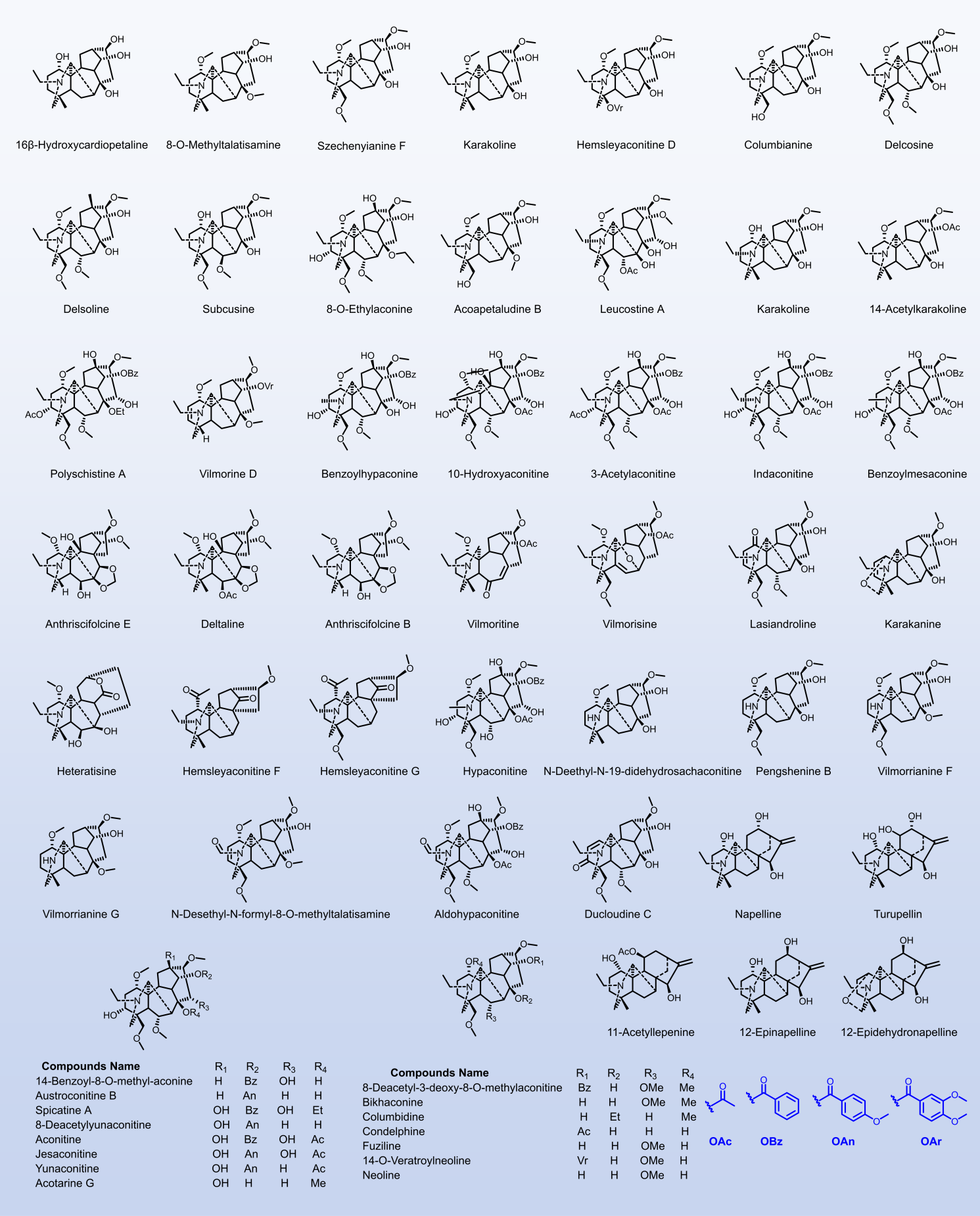


**Supplementary Figure 2.**Chemical structural formulas of diterpenoid alkaloids.

**
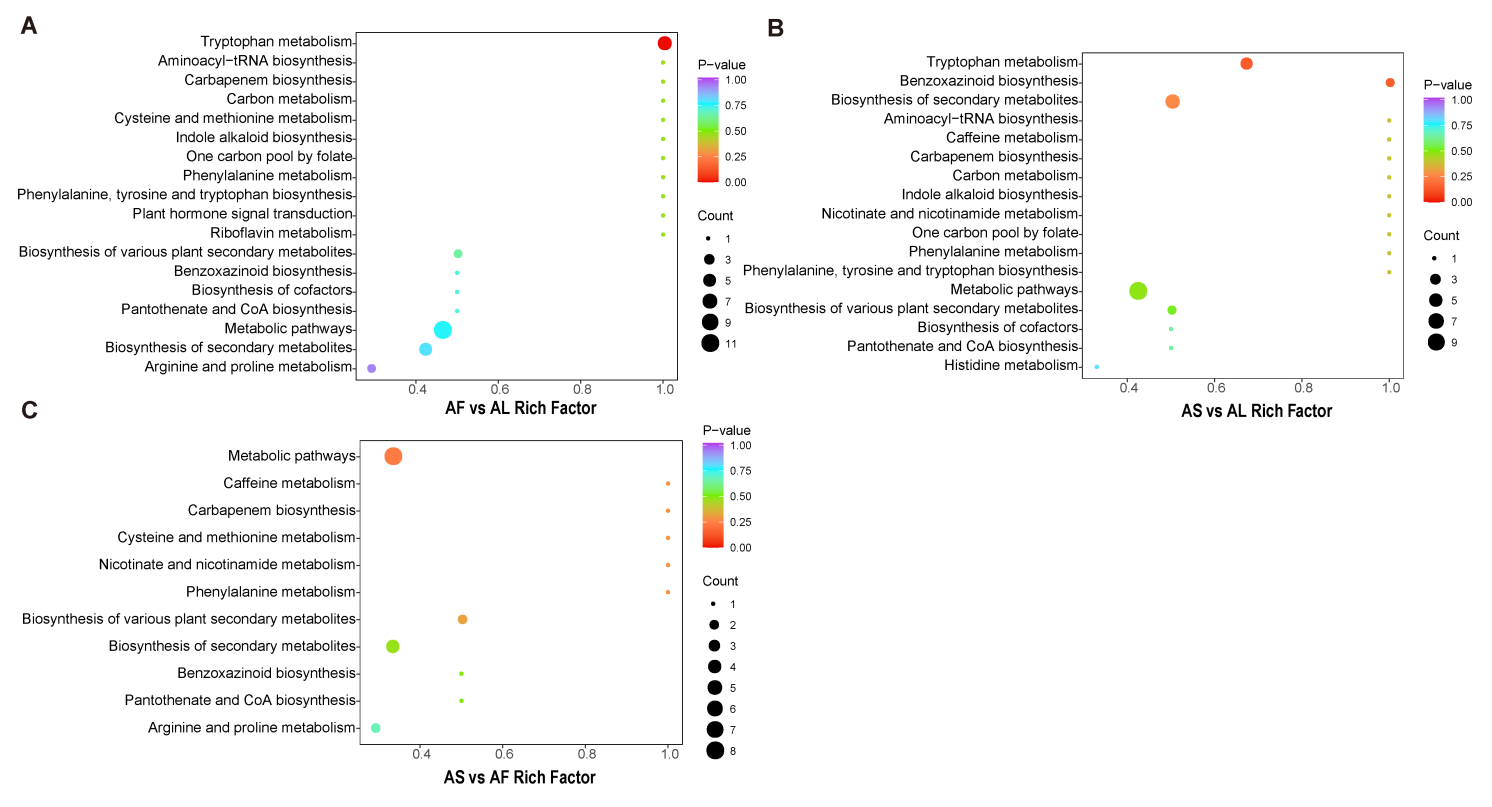
**

**Supplementary Figure 3.** Differential accumulation metabolites (DAMs) KEGG analysis. **(A)** KEGG pathway annotation of of AF vs AL , **(B)** KEGG pathway annotation of AS vs AL, **(C)** KEGG pathway annotation of AS vs AF.


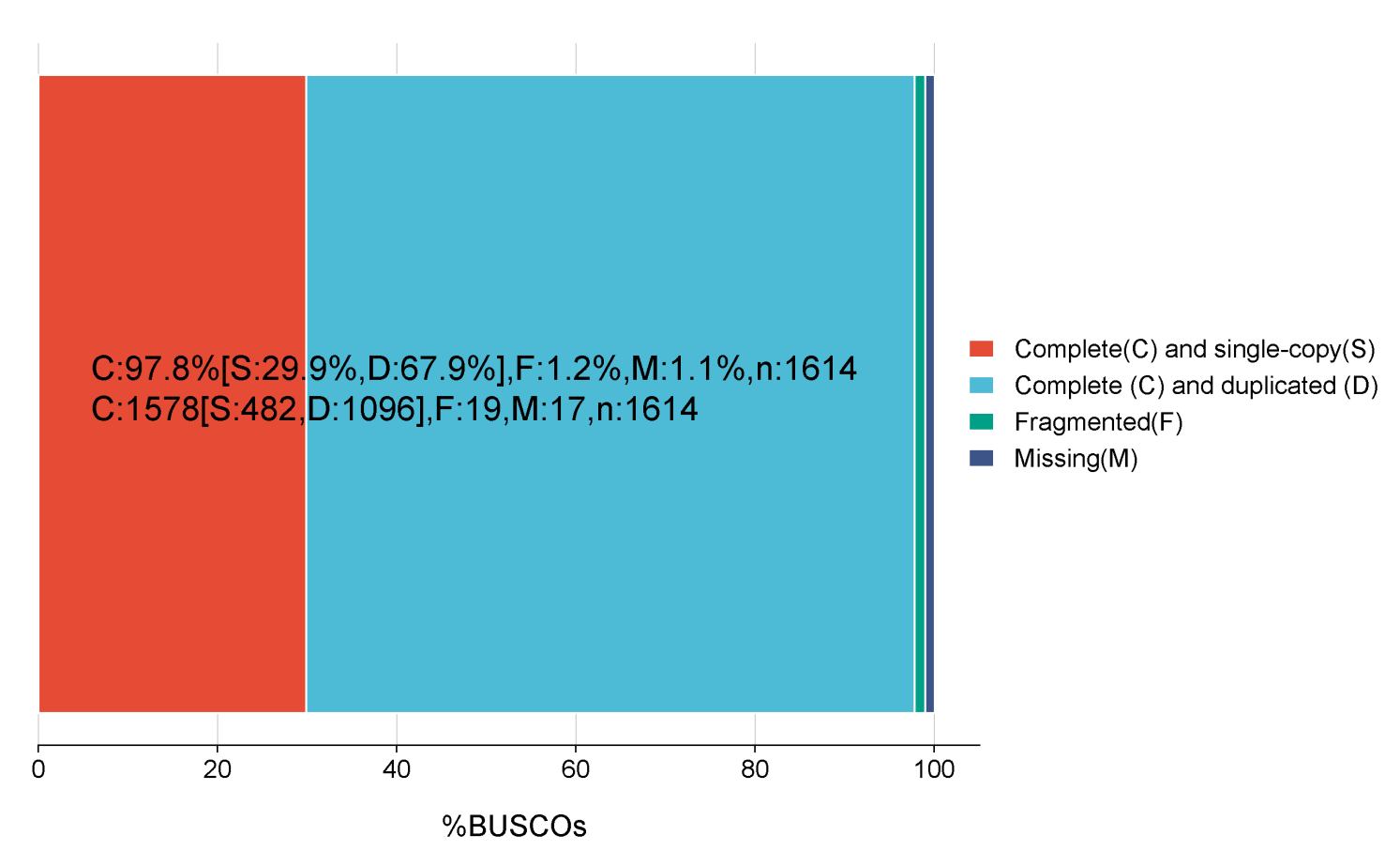


**Supplementary Figure 4.**Assessment of assemblies using Benchmarking Universal Single-Copy Orthologs (BUSCO).


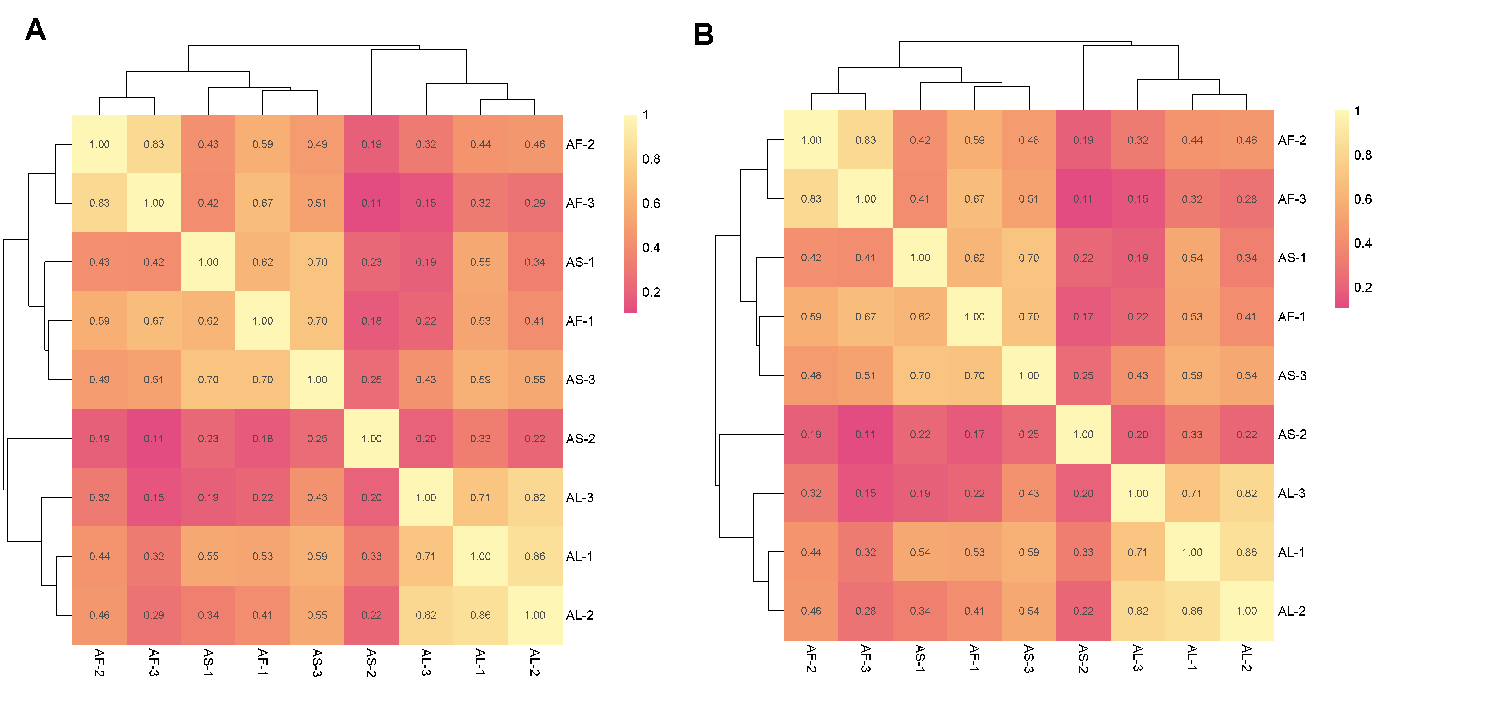


**Supplementary Figure 5.** Heatmap for the correlation coefficient analysis of the replicate groups of samples. **(A)** correlation coefficient analysis of all expressed transcripts in the 9 samples, **(B)** correlation coefficient analysis of after removing the transcripts with low expression levels in these 9 samples.

**
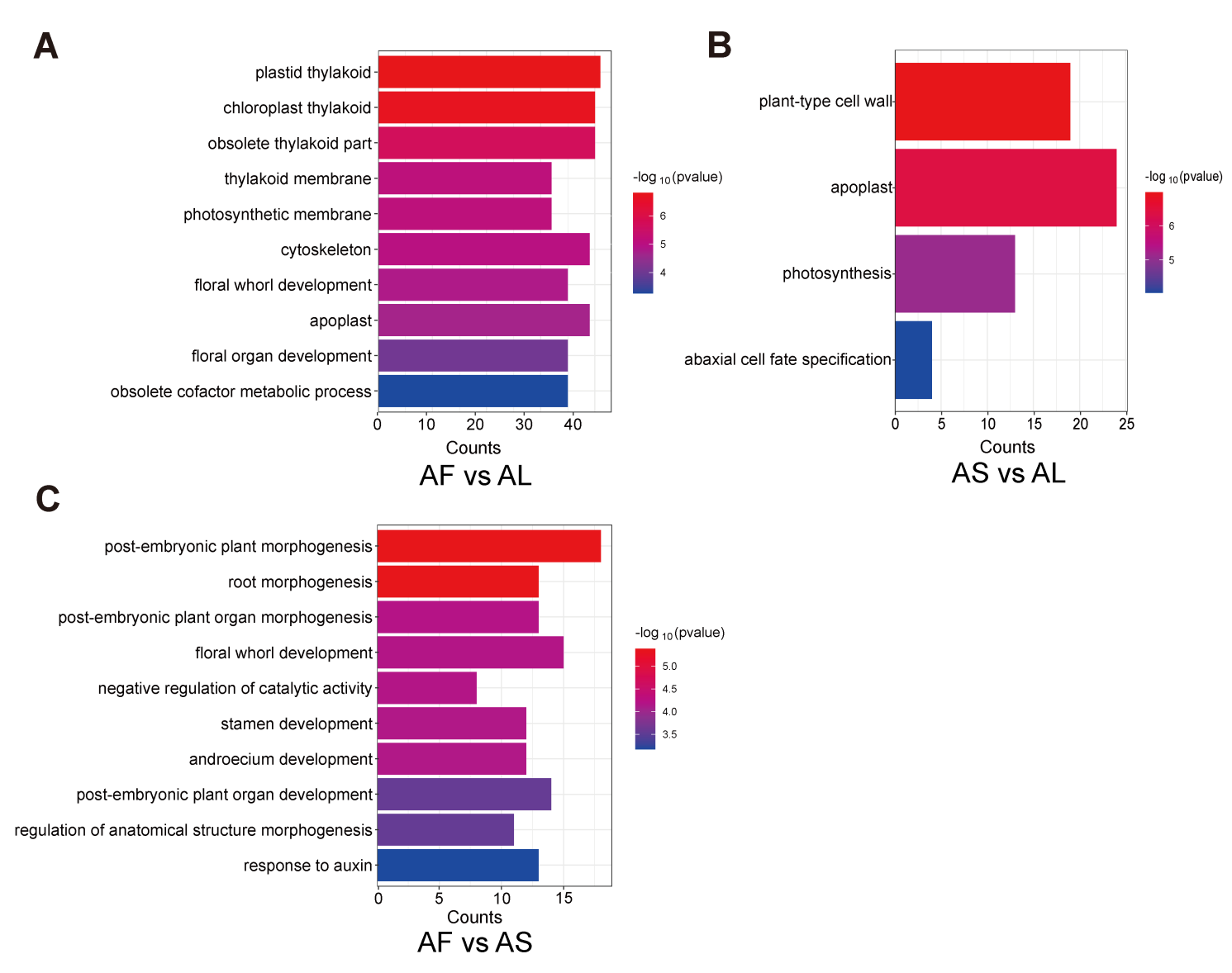
**

**Supplementary Figure 6.** Go function analysis results of differentially expressed genes (DEGs). **(A)** Bar chart of DEGs GO analysis in AF and AL, **(B)** Bar chart of DEGs GO analysis in AS and AL , **(C)** Bar chart of DEGs GO analysis in AF and AS.

**
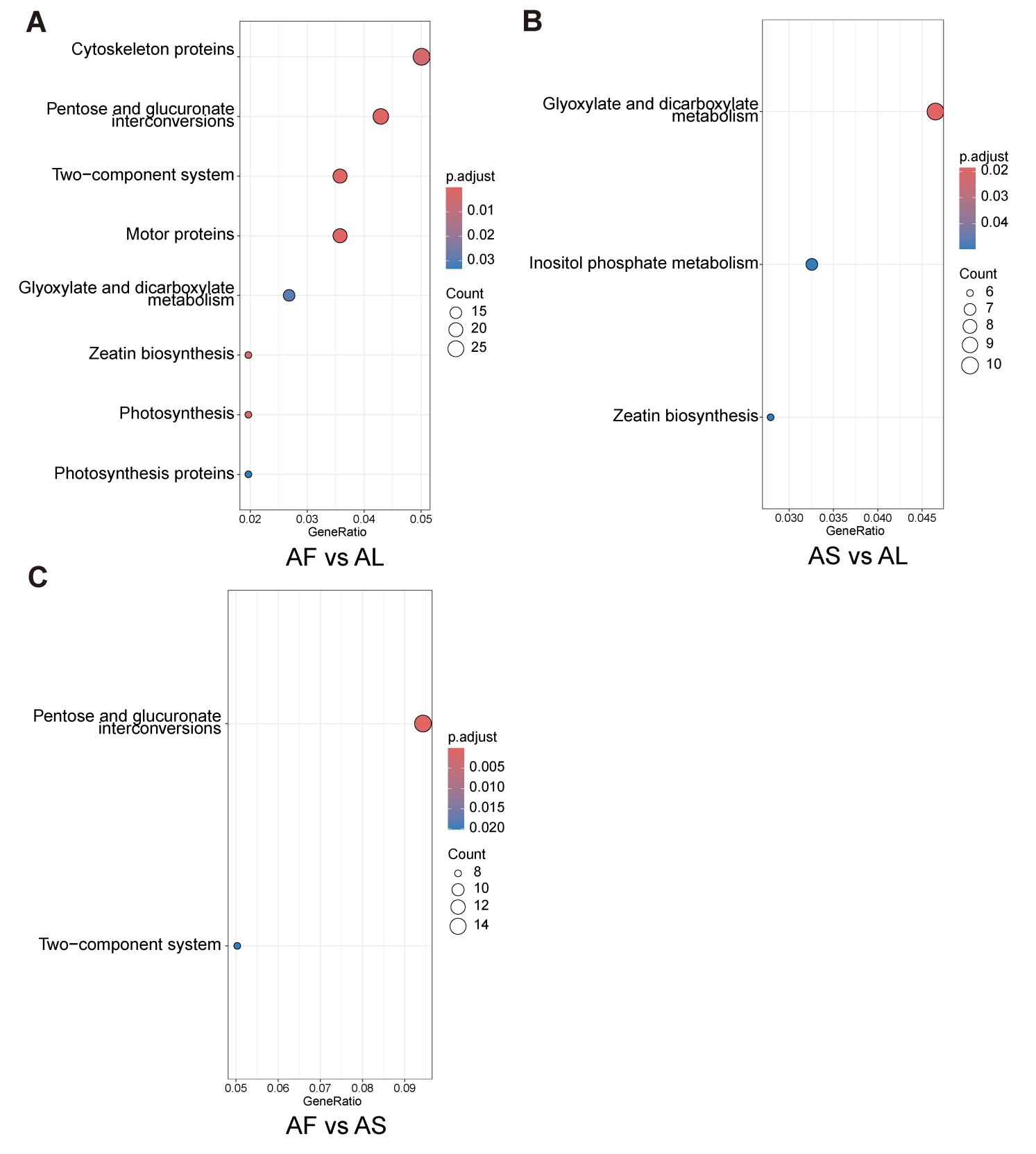
**

**Supplementary Figure 7.** KEGG enrichment map of differentially expressed genes (DEGs). **(A)** Enrichment map of DEGs KEGG analysis in AF and AL, **(B)** Enrichment map of DEGs KEGG analysis in AS and AL, **(C)** Enrichment map of DEGs KEGG analysis in AF and AS.


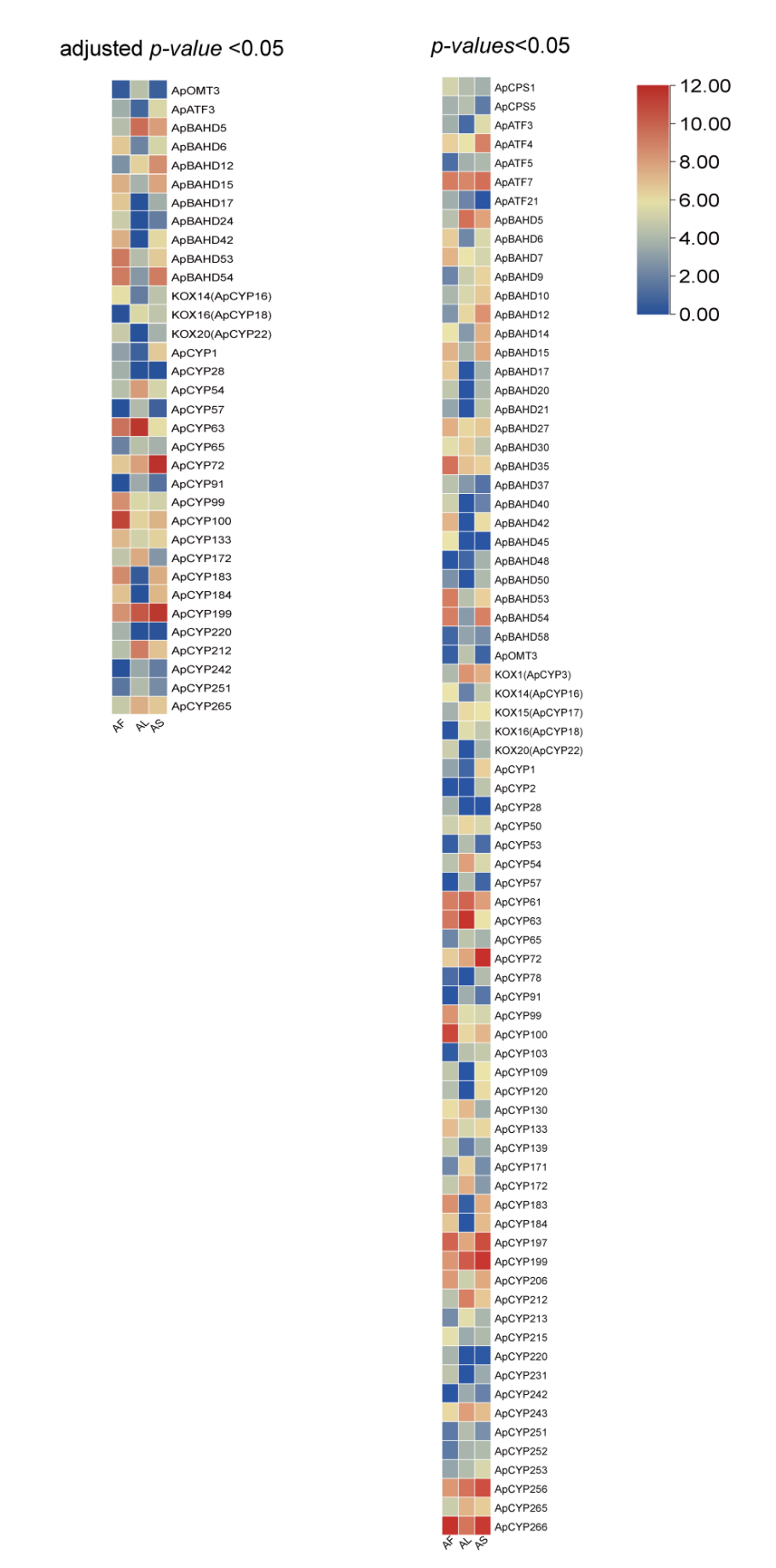


**Supplementary Figure 8.** Heatmap of genes with adjusted *P* <0.05 DEGs and *P* <0.05.The color scale indicates log2 (TPM+1) for the three tissues.

**
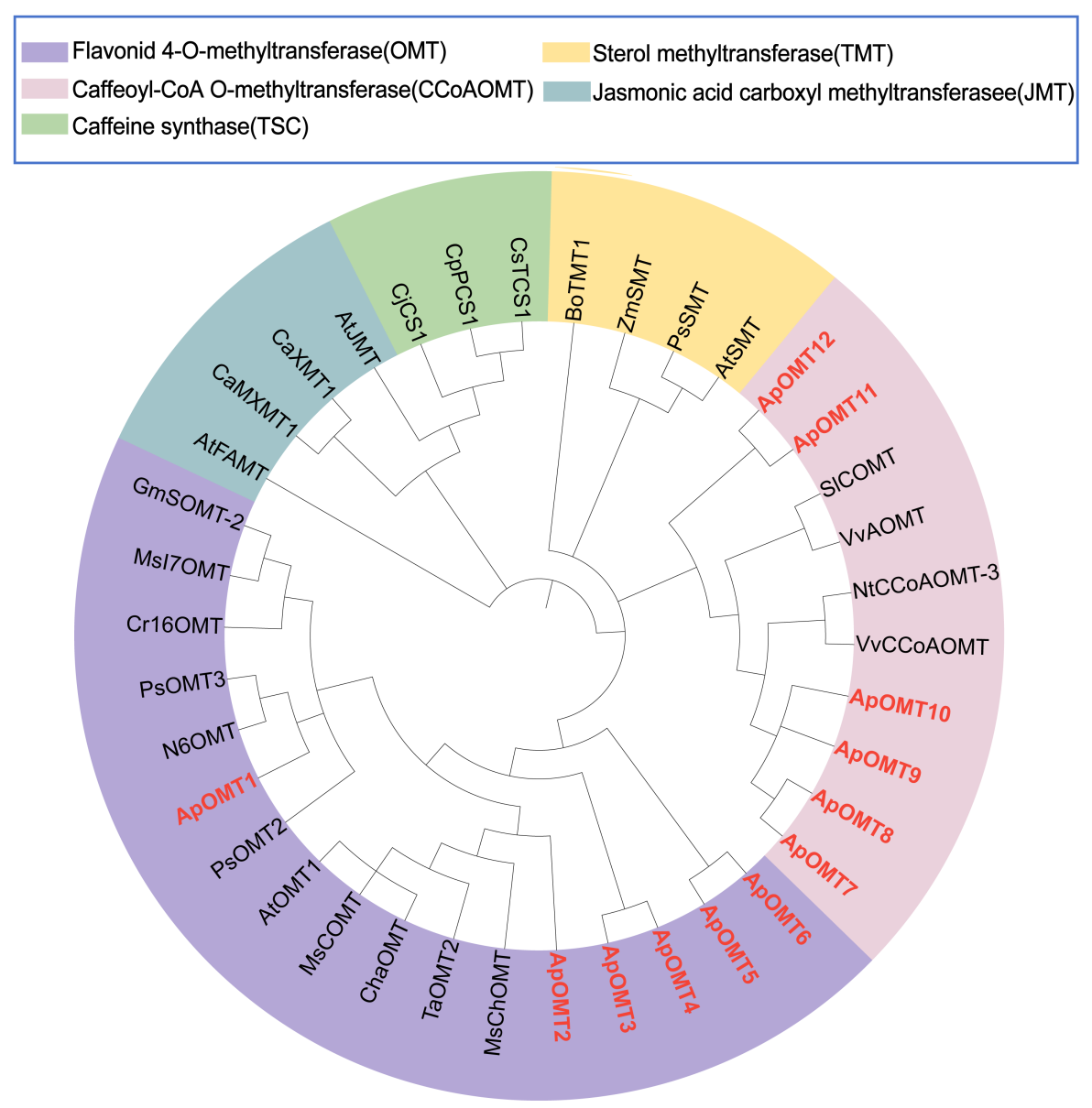
**

**Supplementary Figure 9.** Phylogenetic tree constructed based on the deduced amino acid sequences for the *A.pendulum O*-methyltransferase and other plant OMTs.


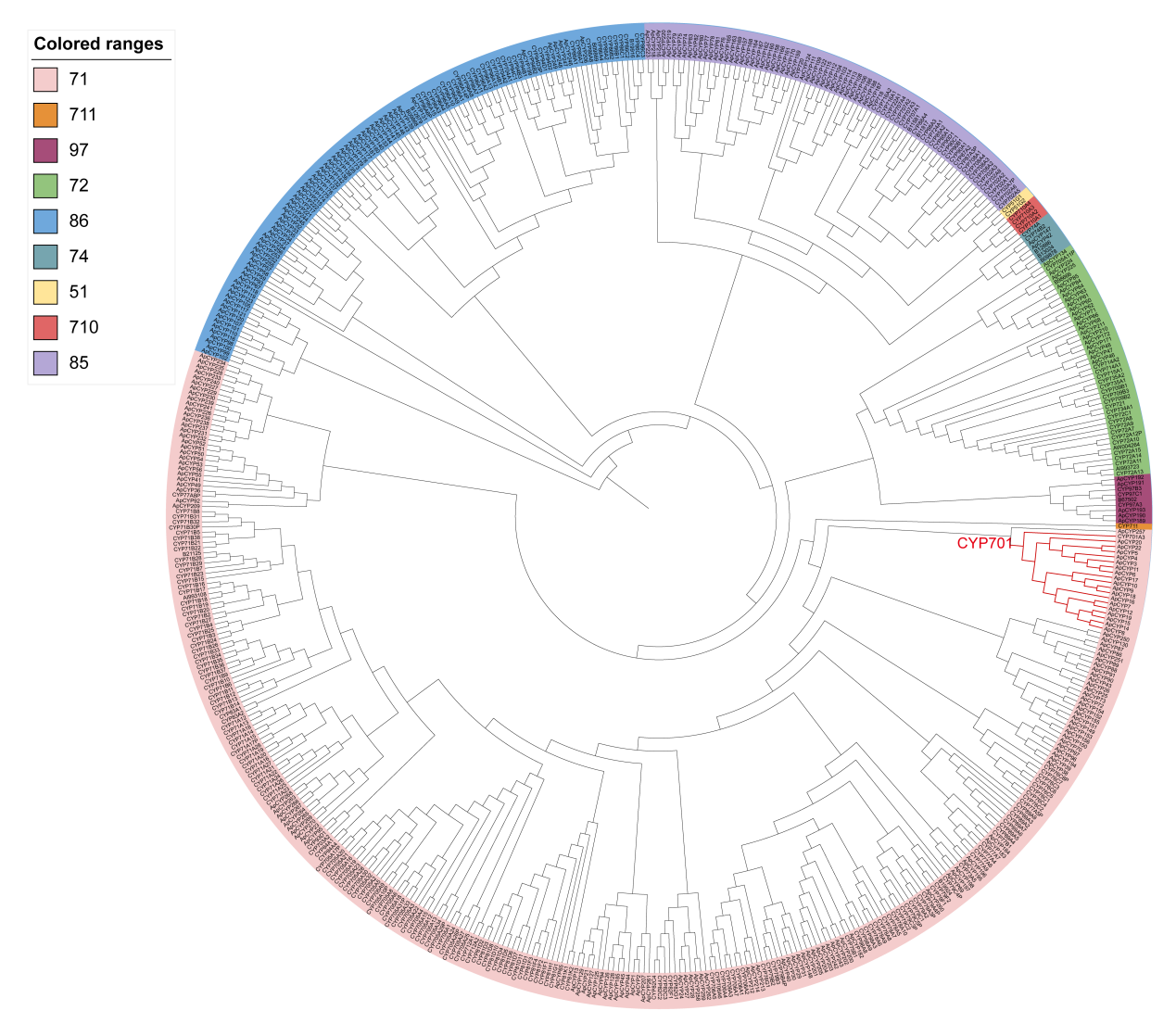


**Supplementary Figure 10.** Phylogeny analysis of the CYP450 gene in *A. pendulum* and *A. thaliana*.


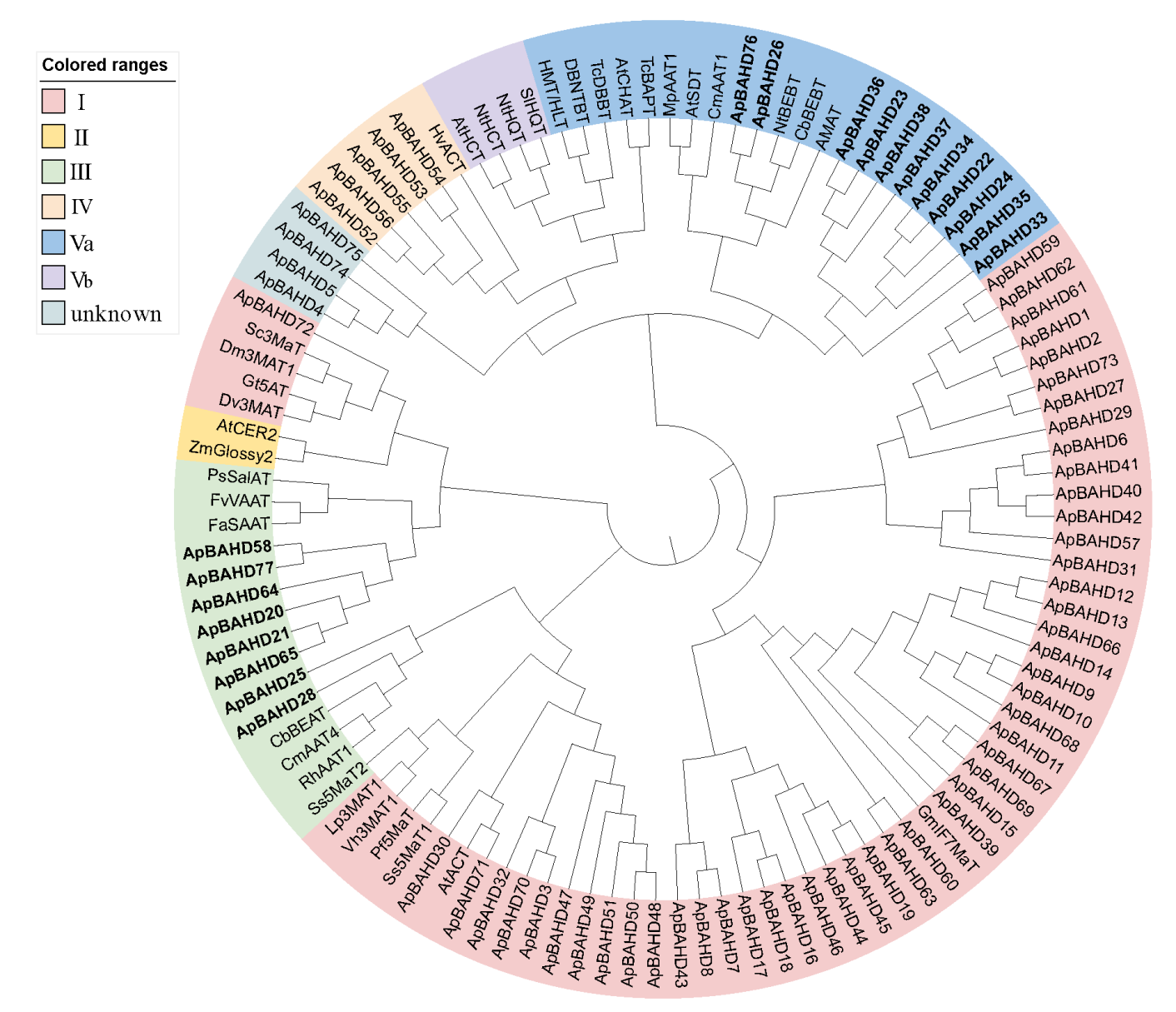


**Supplementary Figure 11.** Phylogenetic relationships among the 77 ApBAHD acyltransferases of *A. pendulum.*
